# A Robust Severe Acute Respiratory Syndrome Coronavirus 2 (SARS-CoV-2)–Specific T- and B-Cell Response Is Associated With Early Viral Clearance in SARS-CoV-2 Omicron-Infected Immunocompromised Individuals

**DOI:** 10.1093/infdis/jiae306

**Published:** 2024-06-06

**Authors:** Magda Vergouwe, Jason J Biemond, Karlijn van der Straten, Lisa van Pul, Gius Kerster, Mathieu Claireaux, Judith A Burger, Karel A van Dort, Neeltje A Kootstra, Marcel Jonges, Matthijs R A Welkers, Mette D Hazenberg, Hessel Peters-Sengers, Marit J van Gils, W Joost Wiersinga, Emma Birnie, Godelieve J de Bree, for the TURN-COVID study group, Wouter Olijhoek, Zakaria Kalmoua, Brent Appelman, Hans L Zaaijer, Frans J van Ittersum, Maarten F Schim van der Loeff, Marije K Bomers, Marie José Kersten, Jarom Heijmans, Marc van der Valk, Mark G J de Boer, Frits R Rosendaal, E Marleen Kemper

**Affiliations:** Center for Experimental and Molecular Medicine, Amsterdam University Medical Center, location AMC, University of Amsterdam, Amsterdam, The Netherlans; Amsterdam institute for Infection and Immunity, Infectious Diseases; Center for Experimental and Molecular Medicine, Amsterdam University Medical Center, location AMC, University of Amsterdam, Amsterdam, The Netherlans; Amsterdam institute for Infection and Immunity, Infectious Diseases; Amsterdam institute for Infection and Immunity, Infectious Diseases; Department of Medical Microbiology and Infection Prevention, Amsterdam University Medical Center, University of Amsterdam, Amsterdam, The Netherlands; Division of Infectious Diseases, Department of Medicine, Amsterdam University Medical Center, University of Amsterdam, Amsterdam, The Netherlands; Amsterdam institute for Infection and Immunity, Infectious Diseases; Department of Experimental Immunology, Amsterdam University Medical Center, University of Amsterdam, Amsterdam, The Netherlands; Amsterdam institute for Infection and Immunity, Infectious Diseases; Department of Medical Microbiology and Infection Prevention, Amsterdam University Medical Center, University of Amsterdam, Amsterdam, The Netherlands; Amsterdam institute for Infection and Immunity, Infectious Diseases; Department of Medical Microbiology and Infection Prevention, Amsterdam University Medical Center, University of Amsterdam, Amsterdam, The Netherlands; Amsterdam institute for Infection and Immunity, Infectious Diseases; Department of Medical Microbiology and Infection Prevention, Amsterdam University Medical Center, University of Amsterdam, Amsterdam, The Netherlands; Amsterdam institute for Infection and Immunity, Infectious Diseases; Department of Experimental Immunology, Amsterdam University Medical Center, University of Amsterdam, Amsterdam, The Netherlands; Amsterdam institute for Infection and Immunity, Infectious Diseases; Department of Experimental Immunology, Amsterdam University Medical Center, University of Amsterdam, Amsterdam, The Netherlands; Department of Medical Microbiology and Infection Prevention, Amsterdam University Medical Center, University of Amsterdam, Amsterdam, The Netherlands; Department of Medical Microbiology and Infection Prevention, Amsterdam University Medical Center, University of Amsterdam, Amsterdam, The Netherlands; Amsterdam institute for Infection and Immunity, Infectious Diseases; Department of Hematology, Amsterdam University Medical Center, University of Amsterdam, Amsterdam, The Netherlands; Department of Hematopoiesis, Sanquin Research, Amsterdam, The Netherlands; Center for Experimental and Molecular Medicine, Amsterdam University Medical Center, location AMC, University of Amsterdam, Amsterdam, The Netherlans; Amsterdam institute for Infection and Immunity, Infectious Diseases; Department of Medical Microbiology and Infection Prevention, Amsterdam University Medical Center, University of Amsterdam, Amsterdam, The Netherlands; Center for Experimental and Molecular Medicine, Amsterdam University Medical Center, location AMC, University of Amsterdam, Amsterdam, The Netherlans; Amsterdam institute for Infection and Immunity, Infectious Diseases; Division of Infectious Diseases, Department of Medicine, Amsterdam University Medical Center, University of Amsterdam, Amsterdam, The Netherlands; Center for Experimental and Molecular Medicine, Amsterdam University Medical Center, location AMC, University of Amsterdam, Amsterdam, The Netherlans; Amsterdam institute for Infection and Immunity, Infectious Diseases; Division of Infectious Diseases, Department of Medicine, Amsterdam University Medical Center, University of Amsterdam, Amsterdam, The Netherlands; Amsterdam institute for Infection and Immunity, Infectious Diseases; Division of Infectious Diseases, Department of Medicine, Amsterdam University Medical Center, University of Amsterdam, Amsterdam, The Netherlands

**Keywords:** SARS-CoV-2, immunocompromised, viral clearance, viral evolution, adaptive immunity

## Abstract

**Background:**

The immunological determinants of delayed viral clearance and intrahost viral evolution that drive the development of new pathogenic virus strains in immunocompromised individuals are unknown. Therefore, we longitudinally studied severe acute respiratory syndrome coronavirus 2 (SARS-CoV-2)–specific immune responses in relation to viral clearance and evolution in immunocompromised individuals.

**Methods:**

Among Omicron-infected immunocompromised individuals, we determined SARS-CoV-2–specific T- and B-cell responses, anti-spike immunoglobulin G (IgG) and IgG3 titers, neutralization titers, and monoclonal antibody (mAb) resistance–associated mutations. The 28-day post-enrollment nasopharyngeal specimen defined early (reverse-transcription polymerase chain reaction [RT-PCR] negative ≤28 days) or late (RT-PCR positive >28 days) viral clearance.

**Results:**

Of 30 patients included (median age, 61.9 [interquartile range, 47.4–72.3] years; 50% females), 20 (66.7%) received mAb therapy. Thirteen (43.3%) demonstrated early and 17 (56.7%) late viral clearance. Patients with early viral clearance and patients without resistance-associated mutations had significantly higher baseline interferon-γ release, and patients with early viral clearance had a higher frequency of SARS-CoV-2–specific B cells at baseline. In non-mAb-treated patients, day 7 IgG and neutralization titers were significantly higher in those with early versus late viral clearance.

**Conclusions:**

An early robust adaptive immune response is vital for efficient viral clearance and associated with less emergence of mAb resistance–associated mutations in Omicron-infected immunocompromised patients. This emphasizes the importance of early SARS-CoV-2–specific T- and B-cell responses and thereby provides a rationale for development of novel therapeutic approaches.

## Teaser

In Omicron-infected immunocompromised patients, early and strong T- and B-cell responses are associated with effective viral clearance and fewer emergences of monoclonal antibody resistance-associated mutations. This highlights the importance of early immune responses, supporting the development of novel therapeutic approaches.

Severe acute respiratory syndrome coronavirus 2 (SARS-CoV-2) has spread across the globe and can result in severe disease with significant morbidity and mortality in vulnerable individuals [1]. Immunocompromised individuals may develop diminished humoral and cellular immune responses following vaccination [2], leading to reduced protection against SARS-CoV-2 (re)infection [3]. Upon infection, persistent polymerase chain reaction (PCR) positivity has often been described in individuals with varying T- and B-cell–related underlying immunocompromised conditions [4–7], putting them at risk for intrahost viral evolution and the emergence of novel immune-evading variants [8–10]. Monoclonal antibodies (mAbs) were initially successfully used to prevent and treat SARS-CoV-2 infections in high-risk patients [11, 12]. However, mAb treatment has been linked to the emergence of resistance mutations [10] and their efficacy drastically decreased against the new variants of concern (VOCs), especially against the Omicron variants and their sublineages [13–15]. Moreover, research has indicated the potential diminishing effect of mAb therapy on the endogenous antibody response [16], which could lead to reduced protection against reinfection. Studies in immunocompetent individuals have elucidated the pivotal role of B cells in both viral clearance and preventing infection through neutralizing antibody formation [17]. Additionally, SARS-CoV-2–specific T cells are activated early in the disease course and their presence is associated with effective viral clearance [18–20], even in the absence of a functional humoral immune response. The delayed viral clearance in immunocompromised individuals is likely a result of deficiencies in both cellular and humoral immunity, but limited data exist on the immunological correlates of viral clearance in immunocompromised patients.

Here, we investigate early longitudinal humoral and cellular immunological determinants of viral clearance and the emergence of mAb resistance–associated mutations and the development of an endogenous immune response in Omicron-infected immunocompromised patients.

## MATERIALS AND METHODS

### Study Population and Design

Samples were collected as part of the TURN-COVID study (ClinicalTrials.gov NCT05195060), a Dutch ongoing multicenter prospective observational cohort study focused on the continuous evaluation of coronavirus disease 2019 (COVID-19) treatments [10, 21]. Immunocompromised adult patients with a reverse-transcription polymerase chain reaction (RT-PCR)–confirmed SARS-CoV-2 infection were enrolled at the Amsterdam University Medical Center between 26 January and 1 November 2022. An immunocompromised state was defined by presence of a hematologic malignancy, immunodeficiency disorder, organ transplant, solid malignancy with systemic treatment, and/or rheumatic diseases with immunosuppressive treatment. Between January and April 2022, high-risk patients received a single 500-mg dose of sotrovimab as recommended by the contemporary Dutch guidelines and were included within 2 days after mAb infusion. A detailed description of the study population and design can be found in the Supplementary Materials.

#### Sample Collection

Nasopharyngeal specimens and blood samples were collected prospectively at inclusion (with a 2-day deviation [range, 0–2]), day 7 (with a 2-day deviation [range, −1 to +1]), 28 (with a 10-day deviation [range, −7 to +10]), and 90 (with a 9-day deviation [range, −7 to +9]). An overview of samples included in each analysis is available in Supplementary Figure 1.

#### Outcomes

The primary outcome was time to SARS-CoV-2 viral clearance, assessed by RT-PCR on day 28 nasopharyngeal specimen. Early viral clearance was defined as *≥*1 RT-PCR with a cycle threshold (Ct) value >34 within 28 days and late viral clearance as *≥*1 RT-PCR with Ct value ≤34 after day 28 [10]. Secondary outcomes included the percentage of SARS-CoV-2–specific T and B cells, interferon gamma (IFN-γ) production by SARS-CoV-2–stimulated peripheral blood mononuclear cells (PBMCs), antibody concentrations measured as anti-spike immunoglobulin G (IgG) and IgG3, pseudovirus neutralization assays, and the emergence of spike protein resistance-associated mutations at position E340 or P337 [22] during treatment.

### Laboratory Assessments

#### SARS-CoV-2 RT-PCR and Variant Sequencing

Nasopharyngeal samples were stored at −80°C. SARS-CoV-2 sequencing and processing were performed as described previously [10]. In short, RNA was extracted using MagNaPure 96 System (Roche Diagnostics, The Netherlands). Whole-genome sequencing (Nanopore sequencing; Oxford Nanopore Technologies) was performed if the Ct value was ≤34. See Supplementary Materials for details.

#### SARS-CoV-2–Specific T- and B-Cell Responses

Day 0 and 28 PBMCs were isolated and stored in liquid nitrogen until further use. The percentage of SARS-CoV-2–specific T cells was determined upon stimulation of PBMCs with a SARS-CoV-2 nucleocapsid- and spike-peptide pool by activation-induced marker assay using flow cytometry. IFN-γ release in the supernatant of the culture was measured by enzyme-linked immunosorbent assay. The percentage of SARS-CoV-2–specific B cells was measured directly ex vivo by flow cytometry after staining PBMCs with a probe mix including the autologous spike protein. Analyses were performed using FlowJo software (BD Biosciences). See Supplementary Materials for details.

#### Serum Antibody and Neutralization Titers

Quantitative determination of serum IgG and IgG3 levels against Omicron BA.1, BA.2, and BA.4/5 spikes on day 0, 7, 28, and 90 was performed using a custom Luminex assay, as described previously [23–25]. Data were expressed as median fluorescence intensity. Omicron BA.1, BA.2, and BA.4/5 pseudoviruses were constructed and neutralization assays were performed using day 7, 28, and 90 serum as described previously [14, 26]. Neutralization titers were expressed as international units per milliliter (IU/mL). Samples with virus neutralization titers <10 IU/mL were defined as having undetectable neutralization. See Supplementary Materials for details.

### Statistical Analysis

Continuous numeric data are presented as median with interquartile range (IQR) and count data as absolute numbers and percentages. Clinical data of early and late viral clearance patients were compared by nonparametric Mann-Whitney *U* test or χ^2^ test where appropriate. Mixed models were used to compare laboratory measurements between patients with early and late viral clearance and patients with and without development of mutations, and to observe changes within groups over time including the interaction term. When assumptions of general mixed models were not met due to the large number of zeroes, zero-inflated mixed models were applied. See Supplementary Materials for details. All statistical analyses were performed using RStudio version 4.2.1. A 2-tailed threshold of *P* < .05 defined statistical significance.

### Ethics

The study was approved by the Amsterdam University Medical Centers medical ethics committee (NL78705.018.21) and conducted in accordance with the Declaration of Helsinki. All study participants provided written informed consent.

## RESULTS

### Patient Characteristics

Of 38 Omicron-infected immunocompromised patients enrolled during the study period, 30 had day 28 nasopharyngeal specimens collected and were included in the present study (Supplementary Figure 1). Fifteen (50%) were female and the median age was 62.8 years (IQR, 47.9–72.1 years; Table 1). Patients were included at a median of 2 days (IQR, 1.0–7.0 days) after SARS-CoV-2–positive RT-PCR and 4.5 days (IQR, 2.0–9.0 days) after symptom onset. Twenty individuals (66.7%) were treated with sotrovimab and enrolled within 2 days after infusion. The mAb-treated patients appeared less severely ill as defined by the COVID-19 World Health Organization (WHO) severity score [27], and had a shorter symptom duration before inclusion (4.0 days [IQR, 2.0–6.5 days]) compared to non-mAb-treated patients (9.5 days [IQR, 8.0–18.0 days]; Supplementary Table 1). Supplementary Table 2 shows detailed patient specifics. Baseline nasopharyngeal specimens were sequenced in 28 of all 30 patients; 18 patients (60.0%, 17 mAb-treated) were infected with Omicron BA.1, 5 (16.7%, 1 mAb-treated) with BA.2, and 5 (16.7%, all non-mAb-treated) with BA.4/5.

**Table 1.** Baseline Demographics, Clinical Characteristics, and Outcome of Patients With Early and Late Viral Clearance

Characteristic	All Patients	Participants by Group, No. (%)	
		Early Viral Clearance	Late Viral Clearance
No. of patients	30	13	17
**Demographics**			
Age, y, median (IQR)	62.8 (47.9–72.1)	61.7 (49.1–72.9)	65.2 (43.5–71.4)
Sex			
Female	15 (50.0)	9 (69.2)	6 (35.3)
Male	15 (50.0)	4 (30.8)	11 (64.7)
BMI, kg/m^2^, median (IQR)	23.7 (21.7–26.8)	24.8 (21.8–26.8)	23.7 (21.1–28.4)
**Clinical characteristics**			
WHO severity score			
2–3 (mild)	20 (66.7)	9 (69.2)	11 (64.7)
4–5 (moderate)	9 (30.0)	4 (30.8)	5 (29.4)
>5 (severe)	1 (3.3)	0 (0.0)	1 (5.9)
SARS-CoV-2 variant^a^			
Omicron BA.1	18 (60.0)	7 (53.8)	11 (64.7)
Omicron BA.2	5 (16.7)	1 (7.7)	4 (23.5)
Omicron BA.4/5	5 (16.7)	3 (23.1)	2 (11.8)
Time between start of symptoms and enrollment, d, median (IQR)	4.5 (2.0–9.0)	4.0 (2.0–9.0)	6.0 (3.0–9.0)
Seronegative (antibodies negative)^b^	20 (66.7)	8 (61.5)	12 (70.6)
Vaccination, fully vaccinated^c^	23 (76.7)	11 (84.6)	12 (70.6)
Specific treatment			
Neutralizing mAbs (sotrovimab)	20 (66.7)	8 (61.5)	12 (70.6)
Anti-IL-6 receptor antagonist	3 (10.0)	0 (0.0)	3 (17.6)
Remdesivir	2 (6.7)	1 (7.7)	1 (5.9)
Convalescent plasma	2 (6.7)	1 (7.7)	1 (5.9)
**Comorbidities**			
Diabetes	3 (10.0)	1 (7.7)	2 (11.8)
Chronic kidney disease	5 (16.7)	0 (0.0)	5 (29.4)
COPD	3 (10.0)	0 (0.0)	3 (17.6)
Cardiovascular disease^d^	10 (33.3)	6 (46.2)	4 (23.5)
Underlying immunosuppressive conditions			
Hematologic malignant neoplasm	15 (50.0)	6 (46.2)	9 (52.9)
Rheumatic disease with immunosuppressive therapy	8 (26.7)	6 (46.2)	2 (11.8)
Stem cell transplant	6 (20.0)	3 (23.1)	3 (17.6)
Solid organ transplant	4 (13.3)	1 (7.7)	3 (17.6)
Solid malignant neoplasm with systemic therapy	2 (6.7)	0 (0.0)	2 (11.8)
Common variable immunodeficiency disorder	2 (6.7)	1 (7.7)	1 (5.9)
**Immunosuppressive medication**			
Any immunosuppressive medication	29 (96.7)	12 (92.3)	17 (100.0)
Corticosteroids	17 (56.7)	8 (61.5)	9 (52.9)
B- or T-cell inhibitors^e^	18 (60.0)	7 (53.8)	11 (64.7)
Rituximab	8 (26.7)	5 (38.5)	3 (17.6)
Chemotherapy^f^	7 (23.3)	3 (23.1)	4 (23.5)
Other^g^	11 (36.7)	7 (53.8)	4 (23.5)
**Outcomes**			
Hospitalized for COVID-19	10 (33.3)	4 (30.8)	6 (35.3)
Length of hospital stay, d, median (IQR)	5.0 (3.5–9.5)	5.5 (4.0–18.3)	6.0 (3.5–10.8)
Oxygen therapy during hospitalization^h^	6 (20.0)	1 (7.7)	5 (29.4)
Intensive care unit admission	2 (6.7)	1 (7.7)	1 (5.9)
90-d mortality	0 (0.0)	0 (0.0)	0 (0.0)

Data are presented as No. (%) unless otherwise indicated.

Abbreviations: BMI, body mass index; COPD, chronic obstructive pulmonary disease; COVID-19, coronavirus disease 2019; IL-6, interleukin 6; IQR, interquartile range; mAbs, monoclonal antibodies; SARS-CoV-2, severe acute respiratory syndrome coronavirus 2; WHO, World Health Organization.

^a^Not sequenced in 2 patients.

^b^In 9 patients, antibodies were not measured. One patient with late viral clearance showed a positive antibody titer.

^c^Fully vaccinated: at least 2 doses and 1 booster dose.

^d^Including medicated hypertension, chronic heart disease, and cerebrovascular disease.

^e^Abatacept, azathioprine, belatacept, cyclosporine, ibrutinib, imatinib, leflunomide, mycophenolate mofetil, mycophenolic acid, pimecrolimus, tacrolimus, teclistamab, or rituximab.

^f^Bendamustine, cisplatin, cyclophosphamide, etoposide, fludarabine, fluorouracil, or capecitabine.

^g^Lenalidomide, methotrexate, hydroxycarbamide, hydroxychloroquine, ruxolitinib, or trastuzumab.

^h^All patients treated with oxygen received corticosteroids according to national guidelines.

Of all participants, 13 (43.3%) had early (RT-PCR–negative result ≤28 days) and 17 (56.7%) late viral clearance (RT-PCR–positive result >28 days). Results of RT-PCR tests on nasopharyngeal swab samples of all follow-up points per subject are provided in Supplementary Table 3 and Supplementary Figure 2. Of all patients with early viral clearance, 11 (84.6%) were fully vaccinated, defined by at least 2 doses and 1 booster dose, compared to 12 of 17 (70.6%) patients with late viral clearance. WHO severity scores and the proportion that received neutralizing mAb therapy were comparable in early and late viral clearance patients. The time between symptom onset and enrollment was 4.0 days (IQR, 2.0–9.0 days) in early and 6.0 days (IQR, 3.0–9.0 days) in late viral clearance patients. Important immunocompromising conditions were hematologic malignancy (50.0%), solid organ transplantation (13.3%), rheumatic disease (26.7%), and immunosuppressive therapy (96.7%). Frequency of hospitalization, length of hospital stay, and administered therapies did not differ significantly between patients with early and late viral clearance.

### Longitudinal Antibody Responses and Neutralizing Capacity of SARS-CoV-2 VOCs

**Figure 1.**
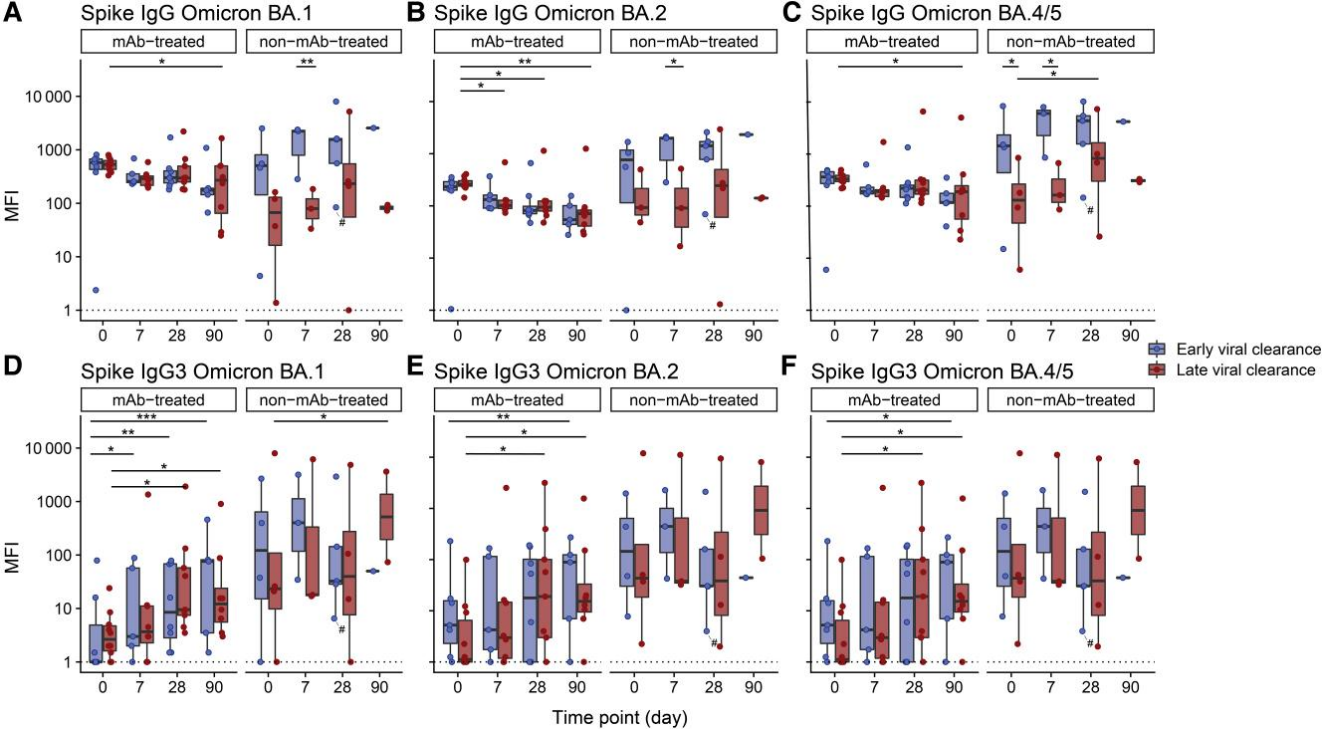
Serum immunoglobulin G (IgG) and IgG3 titer (median fluerescense intensity [MFI]) dynamics in patients with early and late viral clearance against Omicron BA.1 (*A* and *D*), BA.2 (*B* and *E*), and BA.4/5 (*C* and *F*) spikes. Serum levels of 20 monoclonal antibody (mAb)-treated and 10 non-mAb-treated patients at day 0 (n = 25), day 7 (n = 19), day 28 (n = 26), and day 90 (n = 16) are portrayed. Early viral clearance was defined as a negative severe acute respiratory syndrome coronavirus 2 (SARS-CoV-2) reverse-transcription polymerase chain reaction (RT-PCR) ≤28 days and late viral clearance as a positive SARS-CoV-2 RT-PCR >28 days. The hashtag (#) depicts the only sample of a patient treated with convalescent plasma. The dotted line represents the lower limit of detection. The bold line indicates the median, boxes indicate the 25th and 75th percentiles, and whiskers represent 1.5× interquartile range. The y-axis has a logarithmic scale. *P* values are derived from mixed linear models. *P < .05, **P < .01, ***P < .001.

First, using a Luminex bead-based assay, we determined total IgG titers over time on days 0, 7, 28, and 90 (Figure 1). Sotrovimab is an IgG1-based mAb with a half-life of 56.5 days [28]. To differentiate between the endogenous antibody response and the mAb treatment effect, we also measured the serum IgG3 subclass binding. Early or late viral clearance was not associated with a specific SARS-CoV-2 VOC (Table 1). Since mAb-treated patients were mostly infected with Omicron BA.1 (85.0%) and non-mAb-treated patients with BA.2 (40.0%) and BA.4/5 (50.0%), these groups were analyzed separately. Serological status before inclusion in the study was known in 21 patients; 20 were seronegative (19 mAb-treated, 1 non-mAb-treated) and 1 seropositive (non-mAb-treated). In accordance with the pharmacological properties of sotrovimab, in mAb-treated patients, anti-spike BA.1 IgG titers remained stable over 28 days in both the early and late viral clearance patients (Figure 1*A*). After 90 days, decreasing anti-spike binding titers were observed for all SARS-CoV-2 variants in mAb-treated patients, with statistical significance in patients with late viral clearance (*P* = .026 for BA.1; *P* = .005 for BA.2; *P* = .028 for BA.4/5; Figure 1*A–C*). In non-mAb-treated patients, the early viral clearance group showed higher anti-spike IgG titers compared to the late viral clearance group at baseline (*P =* .048 in BA.4/5) and day 7 (*P* = .006 in BA.1, *P* = .017 in BA.2, *P* = .013 in BA.4/5; Figure 1*A–C*). At day 90, no significant difference in anti-spike IgG titers was detected between early and late viral clearance patients for all variants. In all patients, a significant rise in anti-spike BA.1, BA.2, and BA.4/5 IgG3 levels over 90 days, reflective of the endogenous immune response, was observed in both early and late viral clearance patients (Supplementary Figure 3). MAb-treated patients demonstrated a similar significant elevation in anti-spike IgG3 levels for both early (BA.1, *P* < .001; BA.2, *P* = .002; BA.4/5, *P* = .015) and late viral clearance (BA.1, *P* = .044; BA.2, *P* = .010; BA.4/5, *P* = .025; Figure 1*D–F*). We observed an increase in anti-spike IgG3 levels against BA.1 from day 0 to day 90 in non-mAb-treated patients with late viral clearance (*P* = .035; Figure 1*D*). This was not present for Omicron BA.2 and BA.4/5 or early viral clearance patients. We found no significant differences in IgG3 levels between patients with early or late viral clearance in either mAb- and non-mAb-treated patients for all subvariants (Figure 1*D–F*).

**Figure 2.**
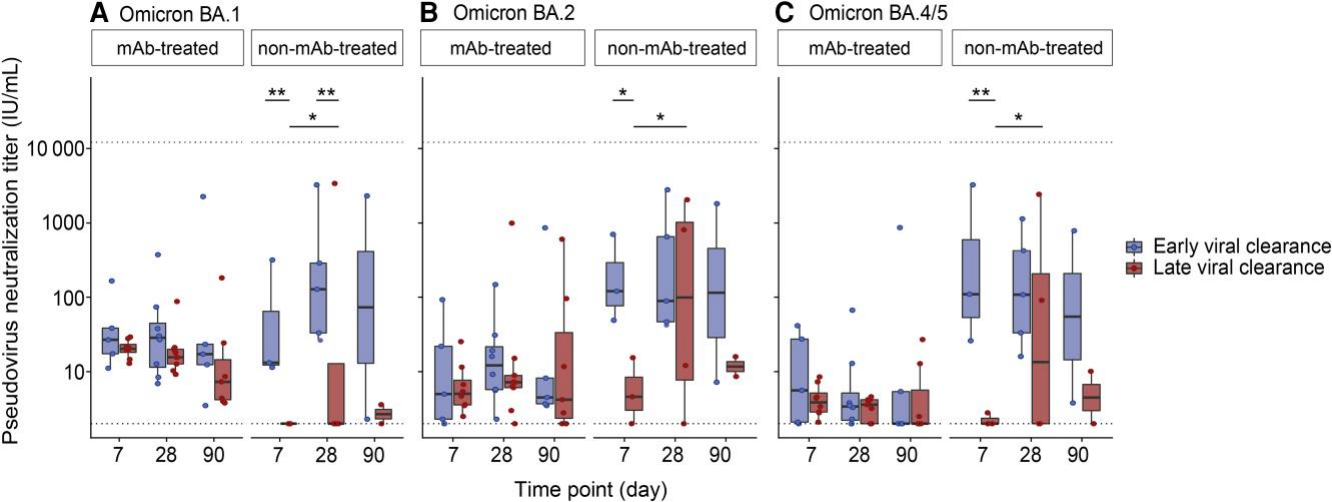
Neutralization half-maximal inhibitory concentration titers (IU/mL) against severe acute respiratory syndrome coronavirus 2 (SARS-CoV-2) Omicron BA.1 (*A*), BA.2 (*B*), and BA.4/5 (*C*) pseudoviruses in 18 monoclonal antibody (mAb)-treated and 9 non-mAb-treated patients at day 7 (n = 19), day 28 (n = 26), and day 90 (n = 16). Early viral clearance was defined as a negative SARS-CoV-2 reverse-transcription polymerase chain reaction (RT-PCR) ≤28 days and late viral clearance as a positive SARS-CoV-2 RT-PCR >28 days. The lower cut-off for neutralization was set at 2 IU/mL and the higher cut-off at 12 150 IU/mL (dotted lines). The bold line indicates the median, boxes indicate 25th and 75th percentiles, and whiskers represent 1.5× interquartile range. The y-axis has a logarithmic scale. *P* values are derived from a mixed linear model. *P < .05, **P < .01.

Additionally, we performed pseudovirus neutralization titers to assess functionality of the detected antibodies. In mAb-treated individuals, neutralization titers did not change significantly over time for all variants and did not differ between early and late viral clearance patients (Figure 2). In non-mAb-treated individuals, similar to IgG dynamics, pseudovirus neutralization titers were significantly lower in late compared to early viral clearance patients at day 7 (*P* = .003 in BA.1, *P* = .011 in BA.2, *P* = .002 in BA.4/5; Figure 2) and day 28 (*P* = .004 in BA.1). For IgG3 levels, we observed no significant differences between early and late viral clearance patients. Serum pseudovirus neutralization showed a significant correlation to IgG binding titers for all Omicron subvariants (Supplementary Figure 4).

### Higher Baseline SARS-CoV-2–Specific B Cells in Early Viral Clearance Patients

**Figure 3.**
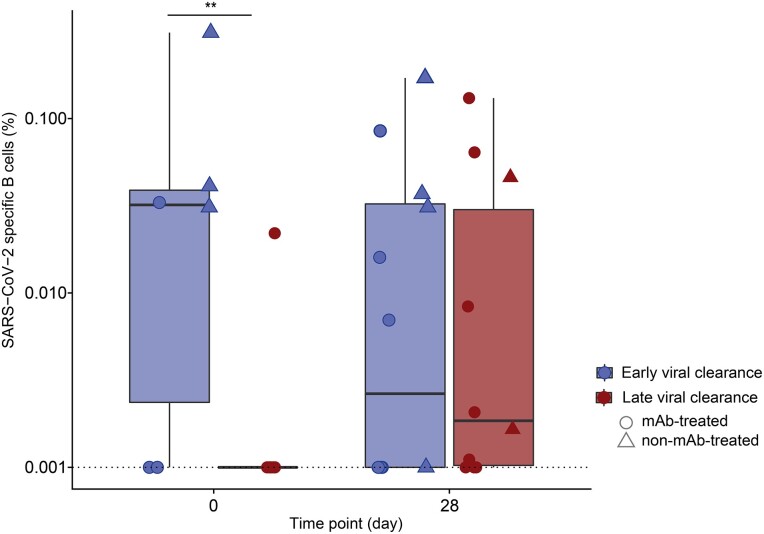
Severe acute respiratory syndrome coronavirus 2 (SARS-CoV-2)–specific B cells over time in patients with early and late viral clearance. The percentage of spike-specific B cells was measured in 13 day 0 and 22 day 28 samples in 24 patients infected with the SARS-CoV-2 Omicron variant. Early viral clearance was defined as a positive SARS-CoV-2 reverse-transcription polymerase chain reaction (RT-PCR) >28 days and late viral clearance as a negative SARS-CoV-2 RT-PCR ≤28 days. Circles represent monoclonal antibody (mAb)–treated patients and triangles represent non-mAb-treated patients. The bold line indicates the median, boxes indicate the 25th and 75th percentiles, and whiskers represent 1.5× IQR. All data points are displayed. Given the prevalence of zeroes in the y-axis value, 0.001 was added to all y-axis data points to account for the logarithmic scale. The dotted line represents the lower limit of detection. Peripheral blood mononuclear cells were stained with a probe mix with 2 colors for the autologous spike protein and measured with fluorescence-activated cell sorting. A zero-inflated mixed model was used in statistical analysis. **P < .01.

Following the observation of development of an endogenous antibody response, next, we determined the association between viral clearance and the SARS-CoV-2–specific B-cell frequency at baseline and day 28 in 18 mAb-treated and 6 non-mAb-treated patients (Figure 3). The SARS-CoV-2–specific B-cell frequency at baseline was significantly higher in patients with early compared to late viral clearance (*P* = .002). Since early and late viral clearance patients had similar disease severity and time since treatment and sampling, these data suggest that this baseline difference is related to the antiviral immune response. By day 28, SARS-CoV-2–specific B-cell frequencies were comparable between early and late viral clearance.

### Early Viral Control Is Associated With SARS-CoV-2–Induced IFN-γ Production of T Cells at Baseline

Next, we assessed SARS-CoV-2–specific T-cell activity by measuring the IFN-γ production by total PBMCs and the percentages of SARS-CoV-2–specific CD4^+^/CD8^+^ T cells. We longitudinally investigated 19 samples at baseline (17 [89.5%] mAb-treated, 7 [36.8%] early viral clearance) and 18 samples at day 28 (15 [83.3%] mAb-treated, 8 [44.4%] early viral clearance).

**Figure 4.**
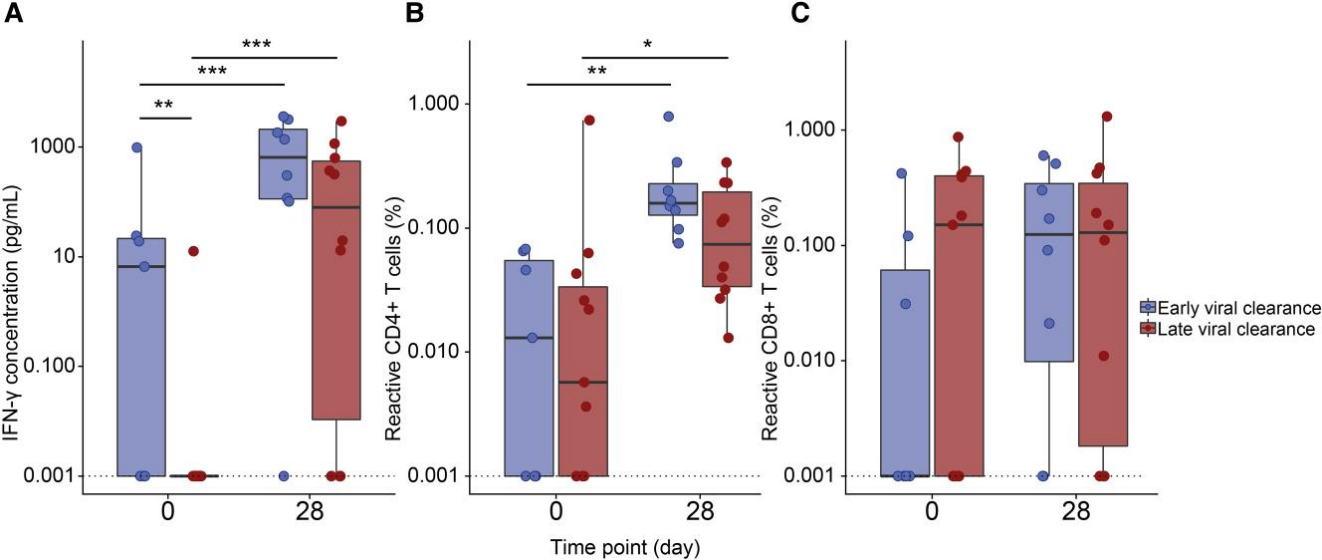
Longitudinal severe acute respiratory syndrome coronavirus 2 (SARS-CoV-2)–specific T-cell immunity in patients with early and late viral clearance. Evolution of spike- and nucleocapsid-specific interferon gamma (IFN-γ) concentration and percentage of CD4^+^ and CD8^+^ T cells were measured at days 0 and 28 in 37 samples of 22 patients infected with the SARS-CoV-2 Omicron variant (Supplementary Figure 1). Early viral clearance was defined as a negative SARS-CoV-2 reverse-transcription polymerase chain reaction (RT-PCR) ≤28 days and late viral clearance as a positive SARS-CoV-2 RT-PCR >28 days. The bold line indicates the median, boxes indicate the 25th and 75th percentiles, and whiskers represent 1.5× interquartile range. Given the prevalence of zeroes in all y-axis values, 0.001 was added to all y-axis data points to account for the logarithmic scale. The dotted line represents the lower limit of detection. *A*, Comparison of IFN-γ concentration as measured by enzyme-linked immunosorbent assay in supernatant of spike- and nucleocapsid-stimulated peripheral blood mononuclear cells between early and late viral clearance patients at days 0 and 28. *B*, Spike-specific reactive CD4^+^ T cells measured by activation-induced marker (AIM) assay in early and late viral clearance patients at days 0 and 28. *C*, Spike-specific reactive CD8^+^ T cells measured by AIM assay in early and late viral clearance patients at days 0 and 28. Zero-inflated mixed models were used in statistical analysis. *P < .05, **P < .01, ***P < .001.

At baseline, patients with early viral clearance exhibited significantly higher IFN-γ concentrations (pg/ml) (median, 6.6 [IQR, 0.0–21.7]) compared to patients with late viral clearance (median, 0.0 [IQR, 0.0–0.0]; *P =* .008; Figure 4). Still, both early and late viral clearance patients demonstrated a significant increase in IFN-γ concentrations (*P* < .001 in early and late) and percentage increase of SARS-CoV-2–specific-CD4^+^ T cells (*P* = .004 in early and *P* = .039 in late) from baseline to day 28. The CD8^+^ T-cell percentage remained stable over time. At both moments, the SARS-CoV-2–specific CD4^+^ and CD8^+^ T-cell percentage did not differ between early and late viral clearance patients. When excluding the 4 non-mAb-treated patients from analyses, similar results were obtained (Supplementary Figure 5). For 10 patients at baseline and 16 patients at day 28, data for both SARS-CoV-2–specific T- and B-cell responses were available. Outcomes were plotted in Supplementary Figure 6.

### A Functional T-Cell Response Protects Against the Emergence of Intrahost Spike Protein Resistance-Associated Viral Mutations

**Figure 5.**
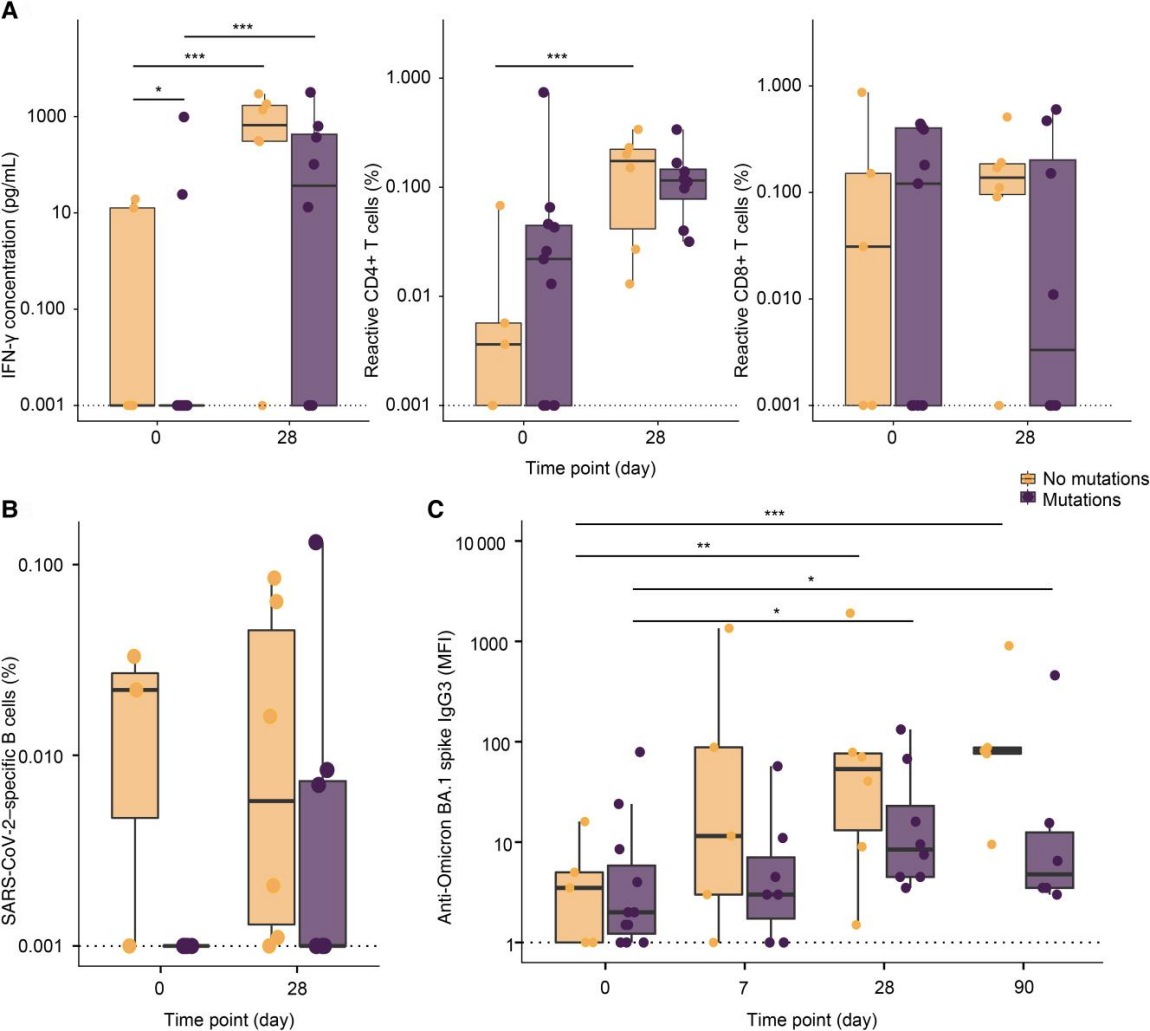
Longitudinal anti-BA.1 spike immunoglobulin G3 (IgG3) and severe acute respiratory syndrome coronavirus 2 (SARS-CoV-2)–specific T- and B-cell immunity in monoclonal antibody (mAb)–treated patients with and without development of resistance-associated mutations. Longitudinal sequencing of nasopharyngeal swabs was performed in 17 mAb-treated patients. The bold line indicates the median, boxes indicate the 25th and 75th percentiles, and whiskers represent 1.5× interquartile range. Given the prevalence of zeroes in the y-axis values in *A* and *B*, 0.001 was added to y-axis data points to account for the logarithmic scale. The dotted line represents the lower assay cut-off. *A*, Comparison of interferon gamma (IFN-γ) concentration as measured by enzyme-linked immunosorbent assay in supernatant of spike- and nucleocapsid-stimulated peripheral blood mononuclear cells and SARS-CoV-2–specific reactive CD4^+^ and CD8^+^ T cells measured by activation-induced marker assay between patients with and without mutations at day 0 and 28. *B*, Comparison of SARS-CoV-2–specific B cells measured with fluorescence-activated cell sorting after staining with a probe mix with 2 colors for the autologous spike protein between patients with and without mutations at day 0 and day 28. *C*, Comparison of IgG3 serum levels (median fluorescence intensity [MFI]) against Omicron BA.1 spike at days 0, 7, 28, and 90 between patients with and without mutation development. *P* values are derived from zero-inflated mixed models. *P < .05, **P < .01, ***P < .001.

As previously described [10], we investigated the development of resistance-associated mutations in sotrovimab-treated patients. Longitudinal nasopharyngeal specimens were available of 17 mAb-treated patients. Of these, 11 (64.7%) developed receptor-binding domain mutations in spike position E340 or P337 within 3–63 days posttreatment. To explore the relationship between adaptive immunity and viral evolution, we compared the IFN-γ production, the percentages of SARS-CoV-2specific CD4^+^/CD8^+^ T cells and B cells, and IgG3 titers between patients with and without development of resistance-associated mutations (Figure 5). We used IgG3 titers against BA.1 since 16 of 17 patients (94.1%) were infected with the BA.1 variant. In patients without mutations, we found higher baseline IFN-γ concentrations (*P* = .011). No significant differences in SARS-CoV-2–specific CD4^+^/CD8^+^ T cells, B cells, or anti-spike BA.1 IgG3 titers were observed between patients with or without mutations.

## DISCUSSION

This study focuses on the role of adaptive immunity in viral clearance in immunocompromised SARS-CoV-2 Omicron-infected individuals treated with or without sotrovimab. In our study, immunosuppressive drug use (96.7%) and (hematologic) malignancies (56.7%) contribute to the significant immunocompromised status, portrayed by the high percentage of SARS-CoV-2 antibody seronegativity (66.7%) at enrollment. In this immunocompromised group, the early functional SARS-CoV-2–specific adaptive immunity, portrayed by higher SARS-CoV-2-induced IFN-γ concentration, higher frequency of SARS-CoV-2–specific B cells, and higher antibody levels, is associated with early viral clearance. Our findings imply a crucial role for both humoral and cellular immunity in viral clearance in the early course of SARS-CoV-2 infection in these specific patients.

In our longitudinal cohort, we show that both functional SARS-CoV-2–specific B and T cells contribute to viral clearance. In addition, the higher percentage of vaccination in the early viral clearance cohort (84.6%) as compared to the late viral clearance cohort (70.6%) could have impacted a stronger SARS-CoV-2–specific T- and B-cell response. The association between delayed viral clearance and impaired adaptive immunity has previously been described in immunocompromised patients [4, 5, 29]. An earlier study conducted in patients with (hematologic) malignancies infected with SARS-CoV-2 wild-type demonstrated that the presence of SARS-CoV-2–specific CD4^+^ T cells in the weeks after infection is associated with a reduced time to viral clearance, using a similar cut-off of 30 days [5]. Additionally, they showed a significant correlation between B-cell depletion, due to anti-CD20 therapy, and persistent PCR positivity [5], which aligned with prior findings by Lee et al [4]. These data are further corroborated by the recent study by Li et al [29], which showed that especially in severely immunocompromised patients (hematologic malignancy and stem cell transplant patients), delayed viral clearance and viral evolution are more pronounced as compared with individuals with autoimmunity and immunosuppressive treatment. We show that a robust early T-cell response, portrayed by IFN-γ release upon stimulation of PBMCs with a SARS-CoV-2 peptide pool, associates with faster viral clearance. Interestingly, we did not observe higher percentages of activated SARS-CoV-2–specific CD4^+^ and CD8^+^ T cells. This discrepancy could be due to the fact that this activated T-cell fraction likely encompasses a larger fraction of SARS-CoV-2–specific T cells, including both IFN-γ–producing and non-IFN-γ–producing cells. Besides the early T-cell response, the fraction of SARS-CoV-2–specific B cells and antibody titers positively associate with earlier viral clearance. This suggests that, especially early in infection, the interplay between humoral and cellular immunity is crucial for viral clearance, for example, through CD4^+^ T-cell co-stimulation of B-cell function. However, due to the limited number of samples with both B- and T-cell data, analysis of the correlation between cellular and humoral immunity in our cohort was not feasible.

As reflected by the clear IgG3 response over 90 days, we showed that despite mAb treatment and their immunocompromised states, mAb-treated patients mount an endogenous immune response. However, we did not find differences in IgG3 levels between mAb-treated patients with early or late viral clearance. Conversely, we did demonstrate higher baseline SARS-CoV-2–specific B-cell percentages in early viral clearance patients and higher IgG and neutralization titers in non-mAb-treated patients with early viral clearance. These disparities between treated and untreated patients might be attributed to the lower disease severity and a shorter duration between symptoms and enrollment in mAb-treated patients. This possibly leads to less pronounced within-group differences due to lower antigen exposure, which is linked to lower endogenous immune responses [30]. Additionally, passive mAb immunization could attenuate the endogenous immune response [16]. Therefore, we cannot conclude whether mAb treatment influences the magnitude of the endogenous antibody response.

In an earlier study, we showed that delayed viral clearance in immunocompromised individuals is associated with development of resistance-associated mutations [10]. Specifically in immunocompromised individuals, selective pressure exerted by mAbs in absence of broader (SARS-CoV-2–specific) T-cell immunity fosters an environment for viruses to successfully evade host immunity [31]. Here, in a larger group of immunocompromised individuals, we found a positive association between elevated IFN-γ levels and early viral clearance and less viral resistance mutations, supporting these suggestions. Given the association we found between elevated IFN-γ levels and early viral clearance, the effect could also be indirect by limiting infection duration and thereby limiting mutation development. In contrast, a study consisting of around 85% immunocompetent individuals found IFN-γ and CD154 expression in spike- and nucleocapsid-stimulated CD4^+^ T cells to be a driver of mutation development [9]. These differences may be explained by the fact that in scenarios of very limited preexisting immunity, some extent of B- and T-cell immune induction during the early weeks after infection positively influences viral clearance and prohibition of viral escape.

In light of the continuing development of resistance against (novel) SARS-CoV-2 therapies [13, 32–34], improved therapeutic strategies remain crucial for immunocompromised individuals. Multiple case series show promising results for combination therapy of several antiviral agents and/or mAbs [35–37]. Another promising approach is virus-specific T-cell (VST) therapy [38], as T cells recognize specific viral epitopes within the spike protein conserved among emerging VOCs [39]. A recent study highlighted the potential effect of VST therapy in 6 immunocompromised individuals with prolonged symptomatic COVID-19 irresponsive to other therapies [40]. In addition, several studies showed promising effects of IFN-γ treatment in SARS-CoV-2–infected immunocompromised individuals with persistent infection [41]. Our study suggests that the window of opportunity, also for prevention of viral escape, may lie within the first weeks following infection. To evaluate optimal indication, safety, and efficacy, these approaches warrant further exploration on a larger scale.

Our study has several strengths. First, to our knowledge, we are the first to evaluate viral clearance and escape together with humoral and cellular immunity in a cohort of mAb- and non-mAb-treated immunocompromised patients in the Omicron era. Second, we provide an in-depth description of longitudinal clinical data. Third, as we exclusively included Omicron-infected individuals, our findings hold relevance for present-day clinical therapies.

Several limitations should be taken into account. First, our cohort is heterogeneous in terms of duration of symptoms, underlying immunosuppressive conditions, and Omicron variants. To address this, we extensively described the clinical data of all patients, offering context to facilitate accurate interpretation of our findings. Second, excluding patients without a day 28 nasopharyngeal specimen collected, due to loss to follow-up or death, potentially introduces selection bias. Third, the small sample size prevented us from correlating clinical outcomes of disease to viral clearance. Also, we did not evaluate Fc receptor function, contributing to viral elimination through, for example, cytokine release and antibody-dependent cellular phagocytosis. Finally, we report a relatively short follow-up duration (28 days) for viral clearance and T- and B-cell responses. Future prospective cohort studies with daily nasopharyngeal swabs could address these limitations, allowing for time-to-event analysis without excluding deceased patients.

In conclusion, we have identified a high prevalence of delayed viral clearance (56.7%) and mAb-resistance-associated mutation development (64.7%) in Omicron-infected immunocompromised patients and show the importance of an early SARS-CoV-2–specific adaptive immune response. Further, we demonstrate the development of an endogenous immune response in immunocompromised patients during neutralizing SARS-CoV-2-mAb treatment. We emphasize the necessity of continued exploration into new therapeutic options, given the rapid emergence of novel VOCs, to ensure the availability of suitable treatments in immunocompromised patients.

## Supplementary Data

Supplementary materials are available at *The Journal of Infectious Diseases* online (http://jid.oxfordjournals.org/). Supplementary materials consist of data provided by the author that are published to benefit the reader. The posted materials are not copyedited. The contents of all supplementary data are the sole responsibility of the authors. Questions or messages regarding errors should be addressed to the author.

## Supplementary Material

jiae306_Supplementary_Data

## Notes

***Acknowledgments.*** The authors would like to thank the research nurses, laboratory technicians, and clinicians at the Amsterdam University Medical Centers (Amsterdam UMC) and all of the patients who participated in this study. The research presented is part of a Dutch cohort study on neutralizing monoclonal antibodies and other antiviral agents against SARS-CoV-2 (TURN-COVID; NCT05195060). We thank the following members of the TURN-COVID study group who were not otherwise compensated: Wouter Olijhoek, MSc, Zakaria Kalmoua, BSc, Brent Appelman, MD, PhD, Hans L. Zaaijer, MD, PhD, Frans J. van Ittersum, MD, PhD, Maarten F. Schim van der Loeff, MD, PhD, Marije K. Bomers, MD, PhD, Marie José Kersten, MD, PhD, Jarom Heijmans, MD, PhD, Marc van der Valk, MD, PhD (all with Amsterdam UMC); Mark G. J. de Boer, MD, PhD, Frits R. Rosendaal, MD, PhD (both with Leiden University Medical Center); and E. Marleen Kemper (Dutch hospital pharmacists’ association, NVZA). We also thank and acknowledge all members of the TURN-COVID study team and all members of the Amsterdam Regional Genomic Epidemiology and Outbreak Surveillance consortium as well as the laboratories participating in open data sharing via GISAID, for sharing contemporary regional SARS-CoV-2 genomic sequences to provide context for the phylogenetic analysis. Data are available upon request.

***Disclaimer.*** The funders had no role in the design and conduct of the study; collection, management, analysis, and interpretation of the data; preparation, review, or approval of the manuscript; or decision to submit the manuscript for publication.

***Financial support*.** This work was supported by the Netherlands Organization for Health Research and Development (ZonMw; number 10430142110001). E. B. is supported by the Netherlands Organization for Scientific Research (NWO) outside the submitted work (VENI grant 09150162210184). W. J. W. is supported by the NWO outside the submitted work (VIDI grant 91716475) and the Talud Foundation (Stichting Talud) for the Amsterdam UMC Corona Research Fund.
