# Supplementary material for: A Robust Severe Acute Respiratory Syndrome Coronavirus 2 (SARS-CoV-2)–Specific T- and B-Cell Response Is Associated With Early Viral Clearance in SARS-CoV-2 Omicron-Infected Immunocompromised Individuals
Source: J Infect Dis. 2024 Jun 6;230(4):e860–71. doi: 10.1093/infdis/jiae306 (PMC11481360; doi:10.1093/infdis/jiae306)
Supplement: jiae306_Supplementary_Data [file jiae306_supplementary_data.pdf]

A Robust SARS-CoV-2-specific T and B Cell Response is Associated with Early Viral Clearance in SARS-CoV-2 Infected Immunocompromised Individuals

Magda Vergouwe[1,2*], Jason J Biemond[1,2*], Karlijn van der Straten[2,3,4], Lisa van Pul[2,5], Gius Kerster[2,3], Mathieu Claireaux[2,3], Judith A Burger[2,3], Neeltje A Kootstra[2,5], Matthijs R A Welkers[3], Mette D Hazenberg[2,6,7], Hessel Peters-Sengers[1], Marit J van Gills[2,3], W. Joost Wiersinga[1,2,4], Emma Birnie[1,2,4#], Godelieve J de Bree[2,4#]; for the TURN-COVID study group **

[*] Both authors contributed equally

[#] Both last authors contributed equally

** Study Group team members are listed in the Acknowledgments

1 Center for Experimental and Molecular Medicine (CEMM), Amsterdam University Medical Center, location AMC, University of Amsterdam, Amsterdam, the Netherlands

2 Amsterdam institute for Infection and Immunity, Infectious Diseases, Amsterdam, the Netherlands

3 Department of Medical Microbiology and Infection Prevention, Amsterdam University Medical Centers, University of Amsterdam, Amsterdam, the Netherlands

4 Division of Infectious Diseases, Department of Internal Medicine, Amsterdam University Medical Centers, University of Amsterdam, Amsterdam, the Netherlands

5 Department of Experimental Immunology, Amsterdam University Medical Centers, University of Amsterdam, Amsterdam, the Netherlands.

6 Department of Hematology, Amsterdam University Medical Centers, University of Amsterdam, Amsterdam, the Netherlands

7 Department of Hematopoiesis, Sanquin Research, Amsterdam, the Netherlands

Corresponding author: Emma Birnie, MD, PhD, Amsterdam UMC - Location AMC, University of Amsterdam, Center for Experimental and Molecular Medicine, Meibergdreef 9, Amsterdam, Netherlands; Phone number: +31205669111; Email: e.birnie@amsterdamumc.nl

This appendix has been provided by the authors to give readers additional information about the work.

Table of content

METHODS

Patient cohort and outcomes Page 3
Laboratory methods Page 3-5
Statistical analysis Page 5-6
Data availability Page 6

REFERENCES Page 6-7

TABLES

Supplementary Table 1. Baseline demographics, clinical characteristics and outcomes of mAb- and non-mAb-treated patients Page 8
Supplementary Table 2. In-depth characteristics of immunocompromised patients with early and late viral clearance Page 9-13
Supplementary Table 3. Overview of all RT-PCR results on nasopharyngeal swab samples per participant Page 14
Supplementary Table 4. Fluorochromes used in FACS analysis Page 15
Supplementary Table 5. Mutations in the spike protein of Omicron BA.2 and Omicron BA.4/5 compared to wild-type spike protein Page 15
Supplementary Table 6. Specifics of the statistical models Page 16

FIGURES

Supplementary Figure 1. Flow diagram of patient inclusion and sampling process Page 17
Supplementary Figure 2. Course of CT values over time per participant Page 18
Supplementary Figure 3. Serum IgG3 titer dynamics in patients with early and late viral clearance against Omicron BA.1, BA.2 and BA.4/5 spike Page 18
Supplementary Figure 4. Correlation between antibody binding and neutralization titers Page 19
Supplementary Figure 5. Longitudinal SARS-CoV-2-specific T cell immunity in mAb-treated patients with early and late viral clearance Page 19
Supplementary Figure 6. Plot of SARS-CoV-2-specific B and T cell responses from patients with combined data available Page 20

METHODS

Patient cohort and outcomes

Samples were collected as part of the TURN-COVID study, a Dutch nationwide prospective observational cohort study. Ambulatory and hospitalized immunocompromised adult patients treated at Amsterdam University Medical Centers, the Netherlands, with an RT-PCR-confirmed SARS-CoV-2 infection between January 26th and November 1st were considered eligible for participation. Immunocompromised status was defined as the presence of a hematologic malignant neoplasm, immunodeficiency disorder, organ transplant, solid malignancy with systemic treatment, and/or rheumatic diseases with immunosuppressive treatment [1]. Sotrovimab received an Emergency Authorization and was provisionally registered for use by the Dutch Working Party on Antibiotic Policy (SWAB) for seronegative patients at high risk of severe COVID-19 and for hospitalized high-risk patients between January and April 2022, during the dominance of the Omicron BA.1 variant in The Netherlands [1]. Patients were included within 2 days after mAb infusion. After April 2022, with the emergence of new Omicron variants, guidelines no longer recommended mAb treatment. Therefore, patients included between April 2022 and November 2022 were either enrolled upon initial contact with their physician or at the time of hospitalization. Only patients with a day-28 nasopharyngeal swab sample available were included.

The primary outcome was time to SARS-CoV-2 viral clearance, assessed by RT-PCR on day 28 nasopharyngeal specimen. Early viral clearance was defined as ≥ one RT-PCR with a cycle threshold (CT) value >34 within 28 days and late viral clearance as ≥ one RT-PCR with CT value ≤34 after day 28 [2]. Secondary outcomes included the percentage of SARS-CoV-2-specific T and B cells, IFN-γ production by SARS-CoV-2-stimulated peripheral blood mononuclear cells (PBCMs), antibody concentrations measured as anti-spike IgG(3), pseudovirus neutralization assays, and the emergence of spike protein resistance-associated mutations. Resistance-associated mutations were defined as mutations at position E340 or P337 during treatment [2, 3], since these positions are associated with the highest reduction of sotrovimab neutralization [4].

Laboratory methods

SARS-CoV-2 whole genome sequencing

SARS-CoV-2 positive materials were transported to the Department of Medical Microbiology, location Academic Medical Center, for full-genome sequencing. RNA extraction was performed on 200 µl of the original patient sample material using a MagNaPure 96 System (Roche Diagnostics, The Netherlands) according to manufacturer's instructions with a total elution volume of 50 µl. Equine arteritis virus (EAV) was added prior to the RNA extraction as an internal extraction control. For cDNA synthesis, the SuperScipt VILO cDNA Synthesis Kit (11754050, ThermoFisher Scientific, The Netherlands) was used according to manufacturer's instructions. Extracted RNA was diluted to an estimated input of 100 copies/reaction in nuclease-free water (AM9939, Ambion, Thermo Fisher Scientific, The Netherlands). In total, 7 µl of diluted RNA solution was combined with 2 µl of 5xVILO reaction mix and 1 µl of 10x SuperScript III enzyme blend to a total reaction volume of 10 µl. cDNA synthesis was performed on a 96-well Biometra thermal cycler for an initial step at 42°C for 30 minutes followed by 5 minutes at 85°C. SARSCoV-2 full genome amplification, adapter ligation and purification were performed using the midnight protocol version 2 (primers purchased via IDT, catalog no: 1007184) [5].

In short, per amplicon pool 10 µl of Q5 Hotstart Hifi 2x master mix, 1 µl of 10 µM midnight primer pool A or B, 5 µl of PCR grade-water (AM9939, Ambion, Thermo Fisher Scientific, The Netherlands) and 4 µl of cDNA was added to a total volume of 20 µl. PCR products of pool A were combined with pool B and subsequently purified using Agencourt AMPure beads (Beckman Coulter, A63881) and eluted in a 20 µl volume according to manufacturer's protocol. RNA quantification was performed using Qubit (Fisher Emergo, product code 10616763) and measured on a Fluostar fluorescence reader (BMG Labtech) according to manufacturer's protocol. After normalization each individual sample was barcoded with the Oxford Nanopore SQK-RBK110.96 rapid barcoding kit. The samples were then pooled together in a single tube and again purified using AMPure XP beads (Beckman Coulter, CA, USA), samples were then processed based on the manufacturer's protocol. Sequencing was performed on R.9.4.1 flowcells (Oxford Nanopore) using high accuracy basecalling. Raw fastq sequencing data was initially mapped to the human genome hg19 to remove contaminant human reads using minimap2 version 2.18-r1015 with default settings3. Unmapped reads were subsequently remapped against reference sequence Wuhan-Hu-1 (Genbank accession number MN908947.3). The resulting sam-file was coverted to bam-file format, sorted and indexed using samtools v.1.104. A consensus sequence was generated using the TrueConsense package (https://github.com/RIVM-bioinformatics/Trueconsense) using settings (-cov 30 -noambig) and manually checked for inconsistencies.

SARS-CoV-2 specific T cell responses

The percentage of SARS-CoV-2 reactive T cells was determined by activation-induced marker (AIM) assay, as described previously [6]. Peripheral blood mononuclear cells (PBMCs) were stimulated with a SARS-CoV-2 nucleocapsid- and spike-peptide pool (JPT Peptide Technologies, Berlin, Germany) or a medium control for 20 hours at 37°C. Cell pellets were then stained for 30 minutes at 4°C for analysis by flow cytometry using the following fluorescent-labeled antibodies: CD3 – BV510, CD8 – Pacific Blue, CD69 – PE-Cy7, CD137 – APC-Fire750, OX40 – PE (BioLegend, San Diego, CA, USA) and CD4 – FITC (BD Biosciences, San Jose, CA, USA). Fluorescence was measured on the fluorescence-activated cell sorting (FACS) Canto II (BD Biosciences). Analysis of marker expression levels was performed using FlowJo Version 10.8.1 (TreeStar, Ashland, OR, USA). CD4 and CD8 reactive T cells were determined by the co-expression of CD137 and OX40 within the CD4 T cell population or the expression of CD137 and CD69 within the CD8 T cell population, respectively. For each sample, the responses specific to the SARS-CoV-2 peptide were determined by subtracting the response in the medium control from the response measured in the peptide pool culture.
IFN-γ release assay was performed to measure SARS-CoV-2-specific T cell responses. In the culture, supernatants of PBMC stimulated with a SARS-CoV-2 nucleocapsid- and spike-peptide pool and a medium control IFN-γ release (pg/ml) was determined by enzyme-linked immunosorbent assay according to manufacturer's protocol (ELISA; Human IFNγ DuoSet ELISA, R&D Systems, Minneapolis, MN, USA). IFN-γ release induced by SARS-CoV-2 was determined by subtracting medium control from the peptide-stimulated culture.

SARS-CoV-2 specific B cell responses

Biotinylated SARS-CoV-2 spike proteins were individually multimerized with fluorescently labeled streptavidin as described previously [7, 8]. Briefly, biotinylated proteins and fluorescently labeled streptavidin were mixed at a 2:1 protein to fluorochrome molar ratio and incubated at 4 °C for 1 h, followed by incubation for at least 30 minutes with 10uM biotin (Genecopoiea) to quenched unbound streptavidin conjugates. The PBMCs were stained with a probe mix including the autologous spike protein in two different colors to minimize background, and the autologous receptor-binding domain (RBD) and the WT spike protein in one color. For the Omicron BA.2 infected individuals, the Omicron BA 4/5 spike protein was used as autologous spike protein, which is antigenically closely related to Omicron BA.2 spike protein [9]. To compensate for the autologous spike protein being added in two different colors (BUV737, BD Biosciences REF:612775 and BV421, Biolegend, REF:405225), the fluorescently labeled wild-type (WT) spike protein (PE-Cy7, Biolegend REF: 405206) was added in a 1:2 ratio. A 1:4 ratio was determined as the optimal ratio for the autologous RBD (AF647, Biolegend, REF: 405237) to also compensate for the RBD being a monomer instead of trimer. The labeled spike and RBD proteins were equimolarly mixed at a final concentration of 45nM before being added to the defrosted PBMC.

The PBMC were defrosted and prepared for staining as described earlier [7]. The cells were stained in Eppendorf tubes with 10uL probe mix and 50ul of staining mix (**Supplementary Table 4**) per 5 million live cells for 30 minutes at 4 °C in the dark and subsequently washed twice in FACS buffer (PBS supplemented with 1 mM EDTA and 2% fetal calf) before taking to the BD LSR Fortessa TM for readout.

To check for assay performance, we included PBMCs of the same SARS-CoV-2 vaccinated individual in each run. The PBMCs of these individuals were stained using the Omicron BA.1 as autologous spike and RBD protein. The compensation was calculated using PMBC of SARS-CoV-2 vaccinated individuals single stained. Analysis was performed using FlowJo version 10.09 software (BD Bioscience). Gating was checked by two different researchers. Samples with a B cell lymphocyte fraction <0.001% of the total lymphocyte population by FACS analysis were excluded from analyses.

SARS-CoV-2 antibody assays

Protein design and purification
Soluble SARS-CoV-2 spike proteins of the variants of concern (VOCs) were produced and purified as described earlier [7, 10]. Mutations in the spike protein of Omicron BA.2 and Omicron BA4/5 compared to WT spike protein (Wuhan Hu-1; GenBank: MN908947.3) are summarized in **Supplementary Table 5**. For the flow cytometry, avi-tagged spike proteins were biotinylated with BirA500 biotin-ligase reaction kit according to manufacturer's instruction (avidity) and purified afterwards using size-exclusion chromatography and stored at -80°C upon further usage.

Protein coupling to MagPlex Microspheres

To measure the binding of IgG and IgG3 to the spike proteins of different VOCs, we covalently coupled pre-fusion stabilized spike proteins to Luminex MagPlex beads using a two-step carbodiimide reaction as previously described [10, 11]. In short, the beads were washed with 100 mM monobasic sodium phosphate pH 6.2 and activated by the addition of Sulfo-N-Hydroxysulfosuccinimide (Thermo Fisher Scientific) and 1-Ethyl-3-(3-dimethylaminopropyl) carbodiimide (Thermo Fisher Scientific) and incubated for 30 minutes on a rotator at room temperature (RT). After washing the activated beads three times with 50 mM MES pH 5.0, the spike proteins were added in a ratio of 75 μg protein to 12.5 million beads and incubated for three hours on a rotator at RT. To block the beads for aspecific binding, we incubated the beads for 30 minutes with PBS containing 2% BSA, 3% fetal calf serum and 0.02% Tween-20 at pH 7.0. Finally, the beads were washed and stored at 4°C in PBS containing 0.05% sodium azide.

Luminex assays

Optimization experiments determined the optimal sera dilution for studying the humoral vaccination response to be 100.000- and 500-fold for IgG and IgG3 binding, respectively. As previously described [11], 50 μL of a bead mixture containing all different spike proteins in a concentration of 20 beads per μL were added to 50 μL of diluted serum and incubated overnight on a rotator at 4°C. The next day, plates were washed with TBS containing 0.05% Tween-20 (TBST) and resuspended in 50 μL of Goat-anti-human IgG-PE (Southern Biotech;2040-09) or Mouse-anti-human IgG3-PE (Southern Biotech; 9210-09). After 2 hours of incubation on a rotator at RT, the beads were washed with TBST and resuspended in 70 μL Magpix drive fluid (Luminex). Read-out of the plates was performed on a Magpix (Luminex). The binding of antibodies was determined as the Median Fluorescence Intensity (MFI) of approximately 50 to 100 beads per well, corrected for background signals by subtracting the MFI of wells containing only buffer and beads. To confirm assay performance, a titration of the serum of one convalescent COVID-19 patient and positive and negative controls were included on each plate. In addition, 15 to 20% of samples of each run were replicated to confirm the results.

Pseudovirus neutralization titers

Pseudovirus construction and neutralization titers were performed as described previously [12, 13]. Shortly HEK293T/ACE2 cells were seeded at a density of 20,000 cells/well in a 96-well plate coated with 50 μg/mL poly-L-lysine one day prior to the start of the neutralization assay. Heat-inactivated sera samples were serially diluted in cell culture medium (DMEM (Gibco), supplemented with 10% FBS, penicillin (100 U/mL), streptomycin (100 μg/mL) and GlutaMax (Gibco)), mixed in a 1:1 ratio with pseudovirus and incubated for 1 hour at 37°C. Subsequently, these mixtures were added to the cells in a 1:1 ratio and incubated for 48 hours at 37°C, followed by a PBS wash and lysis buffer to measure the luciferase activity in cell lysates using the Nano-Glo Luciferase Assay System (Promega) and GloMax system (Turner BioSystems). Relative luminescence units (RLU) were normalized to the positive control wells where cells were infected with pseudovirus without neutralizing Abs or sera. The neutralization titers (IC_{50}) were determined as the serum dilution or antibody concentration at which infectivity was inhibited by 50%, respectively, using a non-linear regression curve fit (GraphPad Prism software version 8.3) and serum dilutions were converted into international units per ml (IU/ml) using the WHO International Standard for anti-SARS-CoV-2 immunoglobulin (NIBSC 20/136). Samples with virus neutralization titers of <10 IU/ml were defined as having undetectable neutralization.

Statistical methods

Two types of mixed models were used to compare all laboratory measurements between groups and to observe changes in each group over time (interaction group * time).

SARS-CoV-2 specific IFN-γ concentrations, CD4+ and CD8+ T cell percentage, and B cell percentage data contained a large number of zeroes. When comparing these values between early and late viral-clearance patients and patients with or without mutations development, assumptions of regular linear mixed models were not met. Therefore, to ensure a proper fit for these right-skewed zero-inflated data, zero-inflated mixed models were applied using the R package glmmTMB (version 1.1.7). Different model properties were tested; timepoint (day 0 or day 28) and group (early versus late viral-clearance and mutations versus no mutations) were checked both separately and combined in the zi.formula specification of the model. Assumptions of all possible models were tested by the dispersion test, Kolmogorov-Smirnoff test, outlier test, Levene's test for homogeneity of variance, and within-group deviation (R package DHARMa version 0.4.6). For specifics of the chosen models for each variable, see **Supplementary Table 6**. In summary, rounding of dependent variables was applied for zero-inflated mixed models to meet the required model assumptions. General linear mixed models were used for all other between groups and over time comparisons after transforming data to a normal distribution using the Box-Cox method [14]. All (co)variances were assumed equal to each other and equivalent to compound symmetry covariance structure,

which is the default setting in R package nlme (version 3.1-162). Time was incorporated as a factor variable in the mixed models to account for nonlinear relationships over time. Assumptions of the mixed models were tested by assessing histograms, skewness and kurtosis of the random intercept. In all models meeting the tested assumptions, the best fit was chosen based on the goodness of fit (lowest Akaike Information Criterion (AIC)) and we corrected for repeated measurement by adding the individual as a random intercept.

Data availability

Data generated in this study were uploaded to GISAID (accession numbers: EPI_ISL_17989859, EPI_ISL_14115175, EPI_ISL_19131402, EPI_ISL_19131404, EPI_ISL_17989860, EPI_ISL_19131406, EPI_ISL_19131407, EPI_ISL_11583041, EPI_ISL_19131408, EPI_ISL_19131409, EPI_ISL_12149948, EPI_ISL_14215762, EPI_ISL_19131400, EPI_ISL_14115214, EPI_ISL_14115215, EPI_ISL_14115216, EPI_ISL_14115217, EPI_ISL_11864888, EPI_ISL_17989858, EPI_ISL_14115172, EPI_ISL_14115204, EPI_ISL_14115168, EPI_ISL_14115201, EPI_ISL_17989857, EPI_ISL_14115164, EPI_ISL_14115165, EPI_ISL_14115184, EPI_ISL_14115192, EPI_ISL_19131420, EPI_ISL_19131395, EPI_ISL_14115178, EPI_ISL_14115179, EPI_ISL_14115180, EPI_ISL_14115181, EPI_ISL_14115207, EPI_ISL_14115210, EPI_ISL_17241376, EPI_ISL_17389140, EPI_ISL_17481180, EPI_ISL_17535664, EPI_ISL_17621930, EPI_ISL_17654831, EPI_ISL_17719162, EPI_ISL_17783358, EPI_ISL_17815222, EPI_ISL_17838506, EPI_ISL_17971223, EPI_ISL_18054466, EPI_ISL_18115956, EPI_ISL_18241707, EPI_ISL_18330957, EPI_ISL_10564135, EPI_ISL_14115166, EPI_ISL_14115169, EPI_ISL_14115194, EPI_ISL_14115202, EPI_ISL_14115208, EPI_ISL_19131410, EPI_ISL_19131411, EPI_ISL_13884353, EPI_ISL_19131413, EPI_ISL_19131412, EPI_ISL_17989855, EPI_ISL_9940086, EPI_ISL_18299377, EPI_ISL_14115176, EPI_ISL_14115177, EPI_ISL_14115173, EPI_ISL_14115193, EPI_ISL_14115206, EPI_ISL_9940085, EPI_ISL_14115170, EPI_ISL_14115196, EPI_ISL_14115203, EPI_ISL_14115205, EPI_ISL_14115209, EPI_ISL_14115211, EPI_ISL_19131414, EPI_ISL_19131415, EPI_ISL_12715944, EPI_ISL_13132087, EPI_ISL_19131418, EPI_ISL_19131416, EPI_ISL_19131417, EPI_ISL_19131419, EPI_ISL_14115212, EPI_ISL_14115213, EPI_ISL_14115218, EPI_ISL_14115191, EPI_ISL_14115200, EPI_ISL_14583608, EPI_ISL_14115171, EPI_ISL_14115195, EPI_ISL_14115167, EPI_ISL_14115190, EPI_ISL_14115198, EPI_ISL_14115199, EPI_ISL_17989856, EPI_ISL_14115174, EPI_ISL_19131396, EPI_ISL_19131399, EPI_ISL_19131397, EPI_ISL_19131398, EPI_ISL_19131403, EPI_ISL_19131401, EPI_ISL_19131405, EPI_ISL_14115164, EPI_ISL_14115165, EPI_ISL_14115184, EPI_ISL_14115192).

REFERENCES

1. Hensgens MPM GE, de Lange DW, Boersma WG, van der Linden PD, Sinha B, de Boer M,. Medical treatment of patients with COVID-19 (SARS-CoV-2 infections). Rotterdam: Dutch Working Party on Antibiotic Policy (SWAB). Available at: https://richtlijnendatabase.nl/richtlijn/covid-19/behandeling/medicamenteuze_behandeling_voor_patienten_met_covid-19.html. Accessed June 5, 2023.
2. Birnie E, Biemond JJ, Appelman B, de Bree GJ, Jonges M, Welkers MRA, Wiersinga WJ. Development of Resistance-Associated Mutations After Sotrovimab Administration in High-risk Individuals Infected With the SARS-CoV-2 Omicron Variant. JAMA **2022**; 328(11): 1104-7.
3. Rockett R, Basile K, Maddocks S, et al. Resistance Mutations in SARS-CoV-2 Delta Variant after Sotrovimab Use. N Engl J Med **2022**; 386(15): 1477-9.
4. Cathcart AL, Havenar-Daughton C, Lempp FA, et al. The dual function monoclonal antibodies VIR-7831 and VIR-7832 demonstrate potent in vitro and in vivo activity against SARS-CoV-2. bioRxiv **2022**: 2021.03.09.434607.
5. Freed NE, Vlková M, Faisal MB, Silander OK. Rapid and inexpensive whole-genome sequencing of SARS-CoV-2 using 1200 bp tiled amplicons and Oxford Nanopore Rapid Barcoding. Biol Methods Protoc **2020**; 5(1): bpaa014.
6. Verburgh ML, van Pul L, Grobben M, et al. Robust Vaccine-Induced as Well as Hybrid B- and T-Cell Immunity across SARS-CoV-2 Vaccine Platforms in People with HIV. Microbiol Spectr **2023**; 11(3): e0115523.

7. Brouwer PJM, Caniels TG, van der Straten K, et al. Potent neutralizing antibodies from COVID-19 patients define multiple targets of vulnerability. Science **2020**; 369(6504): 643-50.
8. Claireaux M, Caniels TG, de Gast M, et al. A public antibody class recognizes an S2 epitope exposed on open conformations of SARS-CoV-2 spike. Nat Commun **2022**; 13(1): 4539.
9. Mühlemann B, Trimpert J, Walper F, et al. Antigenic cartography using variant-specific hamster sera reveals substantial antigenic variation among Omicron subvariants. bioRxiv **2023**: 2023.07.02.547076.
10. van Gils MJ, Lavell A, van der Straten K, et al. Antibody responses against SARS-CoV-2 variants induced by four different SARS-CoV-2 vaccines in health care workers in the Netherlands: A prospective cohort study. PLoS Med **2022**; 19(5): e1003991.
11. Keuning MW, Grobben M, de Groen AC, et al. Saliva SARS-CoV-2 Antibody Prevalence in Children. Microbiol Spectr **2021**; 9(2): e0073121.
12. Caniels TG, Bontjer I, van der Straten K, et al. Emerging SARS-CoV-2 variants of concern evade humoral immune responses from infection and vaccination. Sci Adv **2021**; 7(36): eabj5365.
13. Schmidt F, Weisblum Y, Muecksch F, et al. Measuring SARS-CoV-2 neutralizing antibody activity using pseudotyped and chimeric viruses. J Exp Med **2020**; 217(11).
14. Box GEP, Cox DR. An Analysis of Transformations. Journal of the Royal Statistical Society Series B (Methodological) **1964**; 26(2): 211-52.

TABLES

Supplementary Table 1. Baseline demographics, clinical characteristics and outcomes of mAb- and non-mAb-treated patients

		Participants by group, no (%)	
	**All patients**	**mAb-treated**	**non-mAb-treated**
Number of patients, no	30	20	10
**Demographics**			
Age, median (IQR), years	61.9 (47.4 - 72.3)	59.6 (42.8 - 72.4)	67.5 (62.2 - 71.3)
Sex			
Female	15 (50.0)	11 (55.0)	4 (40.0)
Male	15 (50.0)	9 (45.0)	6 (60.0)
Body mass index [1], median (IQR)	23.7 (21.7 - 26.8)	23.0 (21.1 - 25.3)	25.2 (22.3 - 26.9)
**Clinical characteristics**			
Early viral clearance (RT-PCR negative ≤ 28 days)	13 (43.3)	8 (40.0)	5 (50.0)
WHO severity score			
2-3, Mild	20 (66.7)	17 (85.0)	3 (30.0)
4-5, Moderate	9 (30.0)	2 (10.0)	7 (70.0)
>5, Severe	1 (3.3)	1 (5.0)	0 (0.0)
SARS-CoV-2 Omicron variant [2]			
Omicron BA.1	18 (56.7)	17 (85.0)	1 (10.0)
Omicron BA.2	5 (16.7)	1 (5.0)	4 (40.0)
Omicron BA.4/5	5 (16.7)	0 (0.0)	5 (50.0)
Time between start of symptoms and enrollment, median (IQR), days	5.0 (2.0 - 9.0)	4.0 (2.0 - 6.5)	9.5 (8.0 – 18.0)
Seronegative (antibodies negative) [3]	20 (66.7)	19 (95.0)	1 (10.0)
Vaccination, fully vaccinated [4]	22 (73.3)	16 (80.0)	7 (70.0)
Specific treatment			
Anti IL-6 receptor antagonist	3 (10.0)	2 (10.0)	1 (10.0)
Remdesivir	2 (6.7)	0 (0.0)	2 (20.0)
Convalescent plasma	2 (6.7)	0 (0.0)	2 (20.0)
**Comorbidities**			
Diabetes	3 (10.0)	2 (10.0)	1 (10.0)
Chronic Kidney Disease	5 (16.7)	4 (20.0)	1 (10.0)
Chronic Obstructive Pulmonary Disease	3 (10.0)	2 (10.0)	1 (10.0)
Cardiovascular disease [5]	10 (33.3)	6 (30.0)	4 (40.0)
Underlying immunosuppressive conditions			
Hematologic malignant neoplasm	15 (50.0)	7 (35.0)	8 (80.0)
Rheumatic disease with immunosuppressive therapy	8 (26.7)	7 (35.0)	1 (10.0)
Stem cell transplant	6 (20.0)	4 (20.0)	2 (20.0)
Solid organ transplant	4 (13.3)	3 (15.0)	1 (10.0)
Solid malignant neoplasm with systemic therapy	2 (6.7)	1 (5.0)	1 (10.0)
Common variable immunodeficiency disorder	2 (6.7)	2 (10.0)	0 (0.0)
**Immunosuppressive medication**			
Any immunosuppressive medication	29 (96.7)	19 (95.0)	10 (100.0)
Corticosteroids	17 (56.7)	11 (55.0)	6 (60.0)
B- or T- cell inhibitors [6]	17 (56.7)	12 (60.0)	5 (50.0)
Chemotherapy [7]	7 (23.3)	5 (25.0)	2 (20.0)
Other [8]	11 (36.7)	8 (40.0)	3 (30.0)
**Outcomes**			
Hospitalized for COVID-19	10 (33.3)	3 (15.0)	7 (70.0)
Length of hospital stay, median (IQR), days	5.0 (3.5 - 9.5)	5.0 (4.0 - 18.5)	7.0 (4.0 - 9.5)
Oxygen therapy during hospitalization [9]	6 (20.0)	3 (15.0)	3 (30.0)
Intensive care unit admission	2 (6.7)	1 (5.0)	1 (10.0)
90 day mortality	0 (0.0)	0 (0.0)	0 (0.0)

Abbreviations: mAb: monoclonal antibody; IQR: interquartile range; WHO: World Health Organization
[1] Body mass index is calculated as weight in kilograms divided by height in meters squared;
[2] Not sequenced in two patients;
[3] In 9 patients, antibodies were not measured.
[4] Fully vaccinated: at least two doses and one booster dose;
[5] Including medicated hypertension, chronic heart disease and cerebrovascular disease;
[6] Abatacept, azathioprine, belatacept, cyclosporine, ibrutinib, imatinib, leflunomide, mycophenolate mofetil, mycophenolic acids, pimecrolimus, tacrolimus, teclistamab, or rituximab;
[7] Bendamustine, cisplatin, cyclophosphamide, etoposide, fludarabine, fluorouracil, or capecitabine;
[8] Lenalidomide, methotrexate, hydroxycarbamide, hydroxychloroquine, ruxolitinib, or trastuzumab;
[9] All patients treated with oxygen received corticosteroids according to national guidelines.

Supplementary Table 2. In-depth characteristics of immunocompromised patients with early and late viral clearance

No.	Age range (yrs) & gender	Vaccination status [a]	SARS-CoV-2 antibodies	Co-morbidities	Immuno-suppressive drugs used	Symptoms	Time from start symptoms to study enrollment	Time from first positive PCR to study enrollment	Omicron Sub-variant & Mutation status	Monoclonal antibody treatment	Other treatment	Outcomes
Patients with early viral clearance (negative RT-PCR on nasopharyngeal swab ≤ day 28)												
1	70-80 Female	Complete	Negative	MM, ASCT	IBER, DEX, CYCP	Coughing, headache, sore throat	1 day	2 days	BA.1 Mutation present	Yes		Not hospitalized
2	40-50 Female	Complete	Negative	RA	MTX, RTX, PRED, LEF	Coughing, headache	5 days	1 day	BA.1 Mutation present	Yes		Not hospitalized
3	60-70 Female	Complete	Negative	CVID with GLILD	No	Coughing, fever, dyspnea, rhinorrhea	9 days	9 days	BA.1 Mutation present	Yes		Not hospitalized
4	70-80 Female	Complete	Negative	MM, ASCT	IBER, DEX, CYCP	Rhinorrhea	2 days	1 day	BA.1 Mutation present	Yes		Not hospitalized
5	20-30 Female	Complete	Negative	RA	RTX	Coughing, headache, fatigue, rhinorrhea	2 days	1 day	Not tested	Yes		Not hospitalized
6	80-90 Male	Complete	Negative	RA	RTX, MTX, PRED	Headache	3 days	0 days	BA.1 Mutation present	Yes		Not hospitalized
7	40-50 Female	Complete	Negative	RA	MTX, RTX	Rhinorrhea	1 day	1 day	Not tested	Yes		Not hospitalized
8	40-50 Female	Complete	Negative	SLE, SS	MMF, PRED, HCQ	Sore throat	4 days	4 days	BA.1 Mutation present	Yes		Not hospitalized

No.	Age range (yrs) & gender	Vaccination status[a]	SARS-CoV-2 antibodies	Co-morbidities	Immuno-suppressive drugs used	Symptoms	Time from start symptoms to study enrollment	Time from first positive PCR to study enrollment	Omicron Sub-variant	Monoclonal antibody treatment	Other treatment	Outcomes
Patients with early viral clearance (negative RT-PCR on nasopharyngeal swab ≤ day 28)												
9	50-60 Male	Complete	Unknown	WM	BENDA, RTX	Coughing, fatigue, myalgia, joint pain	2 days	2 days	BA.2 Mutation unknown	No		Not hospitalized
10	60-70 Female	Partial	Unknown	Mantle cell lymphoma, ASCT	DEX	Fever, fatigue	9 days	3 days	BA.1 Mutation unknown	No	DEX, CP	Length of hospital stay (7 days), no O_2, discharged (home)
11	40-50 Female	Complete	Unknown	KTR	MMF, PRED,	Dyspnea, coughing, fever, headache, nausea	12 days	4 days	BA.5 Mutation unknown	No		Length of hospital stay (4 days), no O_2, discharged (home), EDH: KP bacteremia
12	70-80 Male	Unknown	Unknown	EBV-associated lymphoma, PA	PRED, MTX, GOL	Coughing, fever, fatigue, myalgia, joint pain	20 days	2 days	BA.5 Mutation unknown	No	PRED, REM	Length of hospital stay (52 days), O_2 (low flow), discharged (home)
13	70-80 Male	Complete	Unknown	MM	DARA, LEN, DEX	Coughing, fever, myalgia, malaise	29 days	3 days	BA.5 Mutation unknown	No		Length of hospital stay (4 days), no O_2, discharged (home)

No.	Age range (yrs) & gender	Vaccination status[a]	SARS-CoV-2 antibodies	Co-morbidities	Immuno-suppressive drugs used	Symptoms	Time from start symptoms to study enrollment	Time from first positive PCR to study enrollment	Omicron Sub-variant	Monoclonal antibody treatment	Other treatment	Outcomes
Patients with late viral clearance (positive RT-PCR on nasopharyngeal swab > day 28)												
14	40-50 Male	Partial	Negative	DLBCL	DEX, FLU, RTX, POLA, BENDA, CYCP, CART	Myalgia, Joint pain	4 days	0 days	BA.1 Mutation present	Yes		Not hospitalized
15	40-50 Female	Complete	Negative	CF, LTR, KTR, NODAT, HT	TAC, MMF, PRED	No symptoms	0 days	1 day	BA.1 No mutation	Yes		Not hospitalized
16	50-60 Female	Complete	Negative	KTR	TAC, MMF, PRED	Mild symptoms, unspecified	4 days	0 days	BA.1 Mutation present	Yes		Not hospitalized
17	70-80 Male	Complete	Unknown	RA, MI	MTX, PRED	Fever	6 days	1 day	BA.1 Mutation present	Yes		Not hospitalized
18	70-80 Female	Complete	Negative	MM, ASCT, COPD	TEC	Coughing, rhinorrhea	2 days	2 days	BA.1 Mutation present	Yes		Not hospitalized
19	70-80 Male	Complete	Negative	CLL, CKD (3a), AI, prolactinoma,	No	Coughing, fever	8 days	0 days	BA.1 No mutation	Yes	DEX, SAR	Length of hospital stay (3 days), O_2 (low flow), Discharged (rehab)
20	70-80 Male	Complete	Negative	ASCT, DLBCL, CKD (3b), HT	MTX, RTX, HU	Coughing, fever, dyspnea, malaise	9 days	8 days	BA.1 No mutation	Yes	DEX, SAR	Length of hospital stay (32 days), O_2 (Optiflow), Discharged (rehab), EDH: PE, PT

No.	Age range (yrs) & gender	Vaccination status[a]	SARS-CoV-2 antibodies	Co-morbidities	Immuno-suppressive drugs used	Symptoms	Time from start symptoms to study enrollment	Time from first positive PCR to study enrollment	Omicron Sub-variant	Monoclonal antibody treatment	Other treatment	Outcomes
Patients with late viral clearance (positive RT-PCR on nasopharyngeal swab > day 28)												
21	30-40 Female	Complete	Negative	EBV-associated lymphoma	R-CEOP	Headache, rhinorrhea	23 days	23 days	BA.1 No mutation	Yes		Not hospitalized
22	30-40 Male	No	Negative	Myositis	PRED	Sore throat, fever	3 days	2 days	BA.1 Mutation present	Yes		Not hospitalized
23	30-40 Male	Partial	Negative	CVID with GLILD	ABA, PRED	Dyspnea, coughing, sore throat, nausea, malaise	16 days	14 days	BA.1 Mutation unknown	Yes		Length of hospital stay (5 days), O_2 (low flow), discharged (home)
24	60-70 Male	No	Negative	EAC, COPD	T-DXd, CAPE	Diarrhea, sore throat, fever, rhinorrhea, fatigue, loss of taste/smell	4 days	1 day	BA.2 No mutation	Yes		Not hospitalized
25	60-70 Male	Complete	Negative	KTR, HT	PRED, AZA, BELA	Coughing, rhinorrhea, fatigue	2 days	2 days	BA.1 No mutation	Yes		Not hospitalized
26	60-70 Female	Complete	Unknown	AML, ALSCT	PRED, RUX	Dyspnea, malaise, fatigue	2 days	2 days	BA.2 Mutation unknown	No		Not hospitalized
27	70-80 Male	Complete	Positive	CLL, CKD	IBR	Dyspnea, coughing, rhinorrhea	24 days	24 days	BA.2 Mutation unknown	No		Not hospitalized

No.	Age range (yrs) & gender	Vaccination status[a]	SARS-CoV-2 antibodies	Co-morbidities	Immuno-suppressive drugs used	Symptoms	Time from start symptoms to study enrollment	Time from first positive PCR to study enrollment	Omicron Sub-variant	Monoclonal antibody treatment	Other treatment	Outcomes
Patients with late viral clearance (positive RT-PCR on nasopharyngeal swab > day 28)												
28	60-70 Male	Partial	Negative	ALL, HT	CS, IMA	Diarrhea, dyspnea, coughing, nausea	8 days	1 day	BA.5 Mutation unknown	No	DEX, REM, CP	Length of hospital stay (7 days), O_2 (low flow), discharged (home)
29	60-70 Female	Complete	Unknown	Oropharyngeal carcinoma, COPD	CIS, DEX	Rhinorrhea	10 days	7 days	BA.5 Mutation unknown	No		Length of hospital stay (12 days), O_2 (low flow), discharged (nursing home), EDH: COPD exacerbation
30	70-80 Male	Complete	Unknown	Follicular lymphoma, HT	LEN, RTX,	Fever, chest pain, coughing	8 days	1 day	BA.2 Mutation unknown	No		Length of hospital stay (2 days), no O_2, discharged (home)

Abbreviations: DLBCL; Diffuse large cell b-cell lymphoma, CF; cystic fibrosis, LTR; lung transplant recipient, KTR; kidney transplant recipient, NODAT; new onset diabetes after transplantation, HT; hypertension, MM; multiple myeloma, ASCT; autologous stem cell transplant, RA; rheumatoid arthritis, MI: myocardial infarction, CLL; chronic lymphocytic leukemia, CKD; chronic kidney disease, AI; adrenal insufficiency, CVID; common variable immunodeficiency, GLILD; Granulomatous-lymphocytic interstitial lung disease, EAC; esophageal adenocarcinoma, COPD; chronic obstruction pulmonary disease, SLE; systemic lupus erythematosus, SS; Sjögren's syndrome, AML; acute myeloid leukemia, ALSCT; allogenic stem cell transplant, WM; Waldenström macroglobulinemia, ALL; acute lymphoblastic leukemia, PA; psoriatic arthritis, DEX; dexamethasone, FLU; fludarabine, RTX; rituximab, POLA; polatuzumab, BENDA; bendamustine, CYCP; cyclophosphamide, CART; Chimeric antigen receptor T-cell therapy, TAC; tacrolimus, MMF; mycofenolate mofetil, PRED; predniso(lo)ne, IBER; iberdomide, MTX; methotrexate, LEF; leflunomide, TEC; teclistamab, HU; hydroxycarbamide, R-CEOP; rituximab plus cyclophosphamide plus etoposide plus vincristine and prednisone, ABA; abatacept, T-DXd: trastuzumab-deruxtecan, CAPE; capecitabine, AZA; azathioprine, BELA; belatacept, HCQ; hydroxychloroquine, RUX; ruxolitinib, IBR; ibrutinib, CS; cyclosporine, IMA; imatinib, CIS; cisplatin, LEN; lenalidomide, EPC; epcoritamab, GOL; golimumab, SAR; sarilumab, REM; remdesivir, CP; convalescent plasma, O_2; oxygen, EDH; events during hospitalization, KP; Klebsiella pneumoniae, PT; pneumothorax, PE: pulmonary embolism

Supplementary Table 3. Overview of all RT-PCR results on nasopharyngeal swab samples per participant

Patient	Day 0	Day 7	Day 28	Day 90
Patients with early viral clearance				
1	Positive	Positive	Negative	Negative
2	Positive	Positive	Negative	Unavailable
3	Positive	Positive	Negative	Unavailable
4	Positive	Positive	Negative	Positive (new strain)
5	Positive	Positive	Negative	Negative
6	Positive	Unavailable	Negative	Negative
7	Positive	Unavailable	Negative	Unavailable
8	Positive	Positive	Negative	Negative
9	Positive	Positive	Negative	Negative
10	Positive	Unavailable	Negative	Negative
11	Positive	Positive	Negative	Unavailable
12	Positive	Positive	Negative	Unavailable
13	Positive	Negative	Unavailable	Unavailable
Patients with late viral clearance				
14	Positive	Positive	Positive	Positive
15	Positive	Positive	Positive	Unavailable
16	Positive	Positive	Positive	Negative
17	Positive	Positive	Positive	Unavailable
18	Positive	Unavailable	Positive	Positive
19	Positive	Positive	Positive	Negative
20	Positive	Positive	Positive	Positive
21	Positive	Positive	Positive	Positive
22	Positive	Positive	Positive	Negative
23	Positive	Unavailable	Positive	Negative
24	Positive	Positive	Positive	Negative
25	Positive	Positive	Positive	Positive
26	Positive	Positive	Positive	Unavailable
27	Positive	Positive	Positive	Negative
28	Positive	Positive	Positive	Positive
29	Positive	Positive	Positive	Negative
30	Positive	Positive	Positive	Negative

RT-PCR positivity was defined by a cycle threshold (CT) value ≤34.

Supplementary Table 4. Fluorochromes used in FACS analysis

AB	Clone	Manufacturer	Reference
CD38 BB515	HIT2	BD	564498
Via eF780 1/10	N/A	eBioscience	65-0865-14
CD14 eF780	61D3	eBioscience	47-0149-42
CD16 eF780	CB16	eBioscience	47-0168-42
CD3 eF780	UCHT1	eBioscience	47-0038-42
CD20 AF700	2H7	BD	560631
IgD BV785	IA6-2	Biolegend	348242
IgG BV605	G18-145	BD	563246
IgM PE-CF-594	G20-127	BD	562539
CD27 PE	O323	Biolegend	302808
CD19 BUV496	HIB19	BD	741141
CD21 BUV395	B-ly4	BD	740288
**Probe**	**Fluorochrome**	**Manufacturer**	**Reference**
Autologous SARS-CoV-2 RBD	AF647	Biolegend	405237
Autologous SARS-CoV-2 spike	BV421	Biolegend	405225
Wuhan spike	PE-cy7	Biolegend	405206
Autologous SARS-CoV-2 spike	BUV737	BD	612775

Supplementary Table 5. Mutations in the spike protein of Omicron BA.2 and Omicron BA.4/5 compared to wild-type spike protein

BA.2		BA.4	
T19I	Y505H	T19I	Q498R
L24S	D614G	L24S	N501Y
Δ25/27	H655Y	Δ25-27	Y505H
G142D	N679K	Δ69-70	D614G
V213G	P681H	G142D	H655Y
G339D	N764K	V213G	N679K
S371F	D796Y	G339D	P681H
S373P	Q954H	S371F	N764K
S375F	N969K	S373P	D796Y
T376A		S375F	Q954H
D405N		T376A	N969K
R408S		D405N	
K417N		R408S	
N440K		K417N	
S477N		N440K	
T478K		L452R	
E484A		S477N	
Q493R		T478K	
Q498R		E484A	
N501Y		F486V	

Supplementary Table 6. Specifics of the statistical models

Dependent variable	Independent variables	Type of model	Transformation of input variable	Family	Zi formula	Random effect
IFN-γ	VC group, timepoint	Zero-inflated mixed model	Rounded	nbinom2	Day + VC group	Patient ID
	Mutation state, timepoint	Zero-inflated mixed model	Rounded	Truncated_genpois	Day+ Mutation state	Patient ID
CD4+ T cells	VC group, timepoint	Zero-inflated mixed model	*100 and rounded	nbinom1	Day	Patient ID
	Mutation state, timepoint	Zero-inflated mixed model	*100 and rounded	nbinom1	Day+ Mutation state	Patient ID
CD8+ T cells	VC group, timepoint	Zero-inflated mixed model	*10 and rounded	ziGamma	1	Patient ID
	Mutation state, timepoint	Zero-inflated mixed model	*10 and rounded	beta_family	1	Patient ID
B cells	VC group, timepoint	Zero-inflated mixed model	*100 and rounded	nbinom1	Day	Patient ID
	Mutation state, timepoint	Zero-inflated mixed model[1]	*100 and rounded	nbinom2	Day+ Mutation state	Patient ID
Anti-spike titers	VC group, treatment group and timepoint	General mixed model	BoxCox	N.A.	N.A.	Patient ID
	Mutation state, timepoint	General mixed model	BoxCox	N.A.	N.A.	Patient ID
Neutralization titers	VC group, treatment group and timepoint	General mixed model	BoxCox	N.A.	N.A.	Patient ID

Abbreviations: VC: viral clearance.

[1] In three occasions the zero-inflated model did not converge properly. Therefore, Man-Whitney *U* test was used for statistics in these occasions.

FIGURES

Supplementary Figure 1. Flow diagram of patient inclusion and sampling process

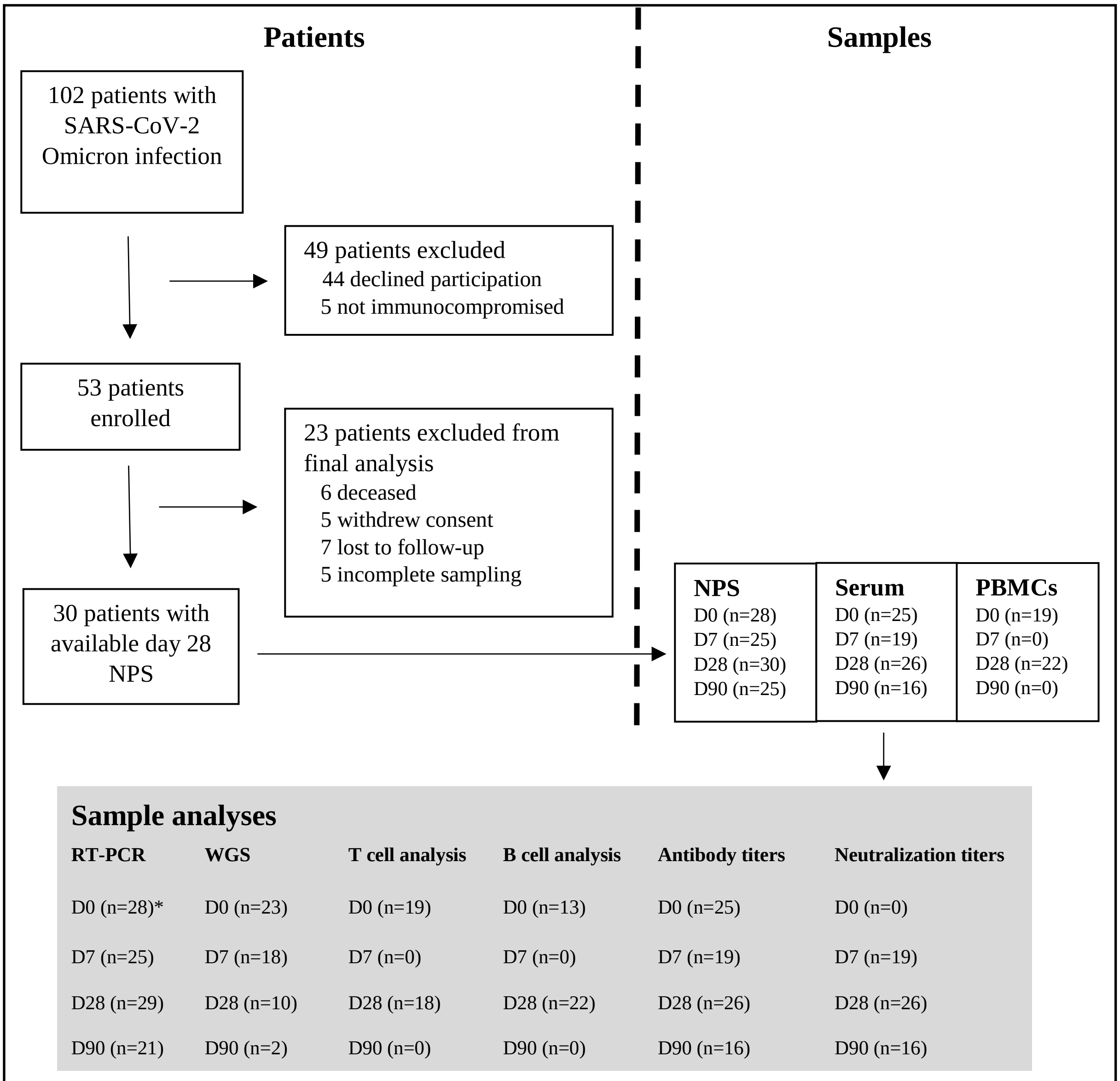

Supplementary Figure 1. Flow diagram of the patient inclusion and sampling process. Abbreviations: NPS: nasopharyngeal swab; PBMCs: peripheral blood mononuclear cells; RT-PCR: reverse transcription-polymerase chain reaction; WGS: whole-genome sequencing.
* Two patients tested SARS-CoV-2 positive in an external laboratory facility

Supplementary Figure 2. Course of CT values over time per participant

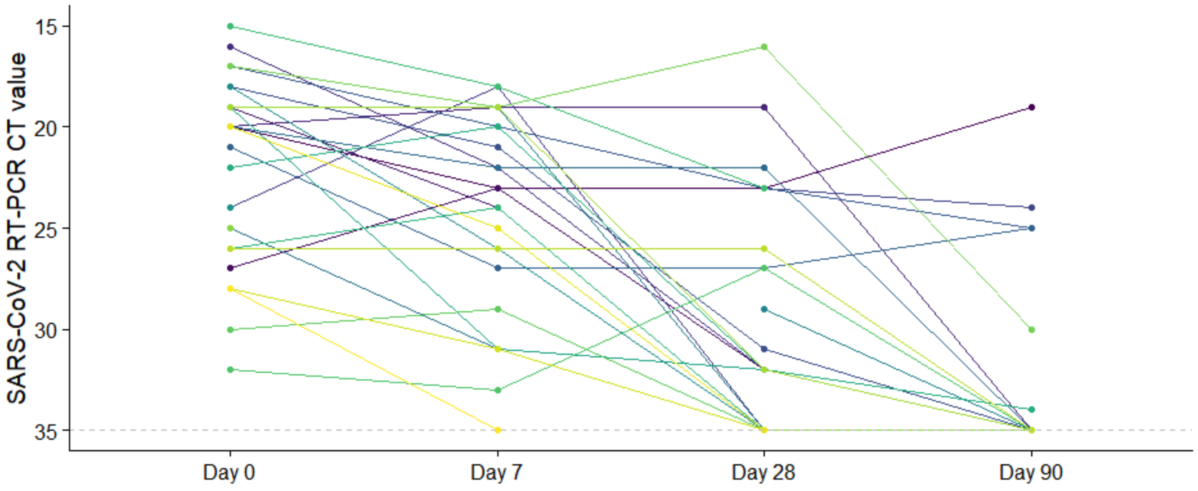

Supplementary Figure 2. Colored dots and lines represent the CT-values of RT-PCRs on nasopharyngeal swab samples for each participant in the total study cohort (n=30). CT-value 35 was reported for negative RT-PCRs (grey dotted line). The Y-axis is inversed. We display 28 day 0 samples, 25 day 7 samples, 29 day 28 samples and 21 day 90 samples. Two patients tested SARS-CoV-2 positive in an external laboratory facility, therefore day 0 CT values were not available for two patients. Abbreviations: CT = cycle threshold.

Supplementary Figure 3. Serum IgG3 titer dynamics in patients with early and late viral clearance against Omicron BA.1, BA.2 and BA.4/5 spike

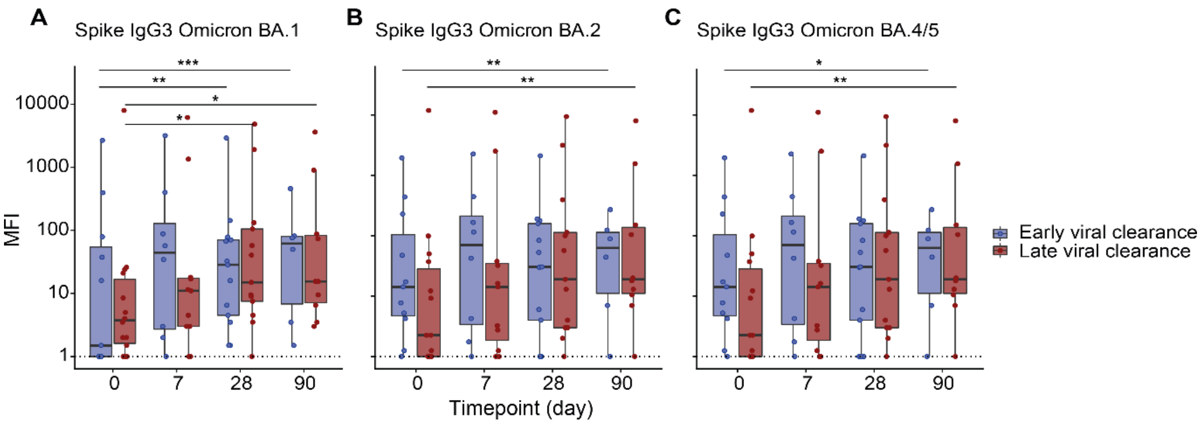

Supplementary Figure 3. Dynamics of IgG3 serum levels (MFI) against spike of Omicron BA.1 (**A**), BA.2 (**B**) and BA.4/5 (**C**) in 30 patients at day 0 (n=25), day 7 (n=19), day 28 (n=26) and day 90 (n=16). Blue represents early viral-clearance patients (negative SARS-CoV-2 RT-PCR ≤28 days) and red late viral-clearance patients (positive SARS-CoV-2 RT-PCR >28 days). The dotted line represents the lower assay cut-off. The bold line indicates the median, boxes indicate 25th and 75th percentiles and whiskers represent 1.5x IQR. Y-axis outcomes were log-transformed. P values are derived from a mixed linear model. * *P*<0.05, ***P*<0.01, ****P*<0.001. Abbreviations: MFI: mean fluorescent intensity; IQR: Interquartile range.

Supplementary Figure 4. Correlation between antibody binding and neutralization titers

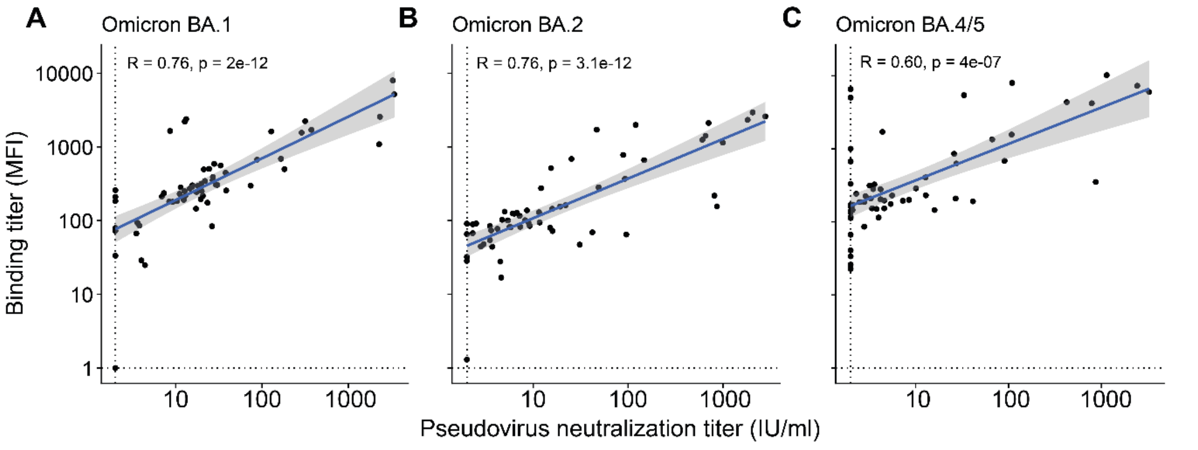

Supplementary Figure 4. Correlation between Omicron BA.1 (A), BA.2 (B) and BA.4/5 (C) binding titers (MFI) of IgG and pseudovirus neutralization titers in half-maximum inhibitory concentration (IC50) (IU/ml). Spearman rank correlation coefficient and p-value are reported. Abbreviations: MFI: mean fluorescent intensity.

Supplementary Figure 5. Longitudinal SARS-CoV-2-specific T cell immunity in mAb-treated patients with early and late viral clearance

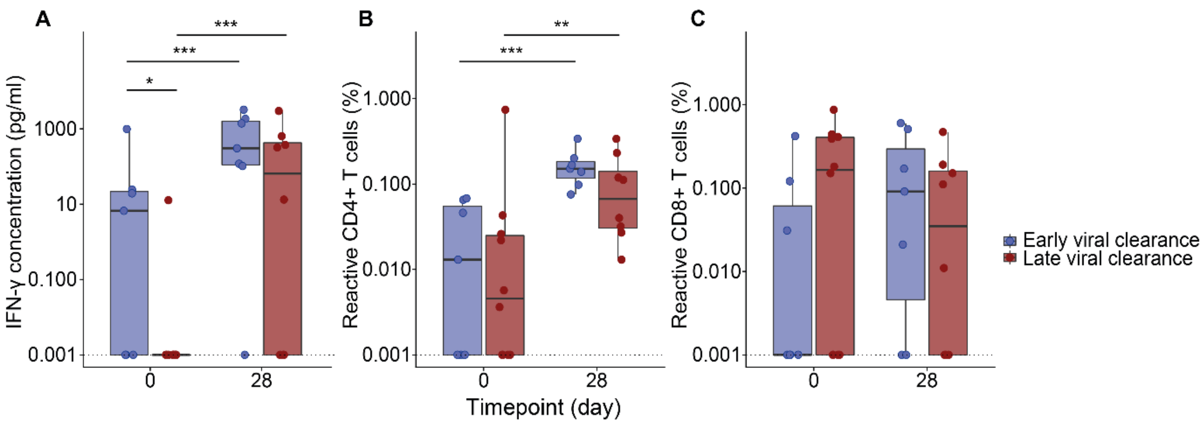

Supplementary Figure 5. Evolution of spike- and nucleocapsid-specific IFN-γ concentration, and percentage of CD4+ and CD8+ T cells were measured at day 0 and 28 in a subset of 32 samples of 18 mAb-treated patients infected with the SARS-CoV-2 Omicron variant. Blue represents early viral-clearance patients (negative SARS-CoV-2 RT-PCR ≤28 days) and red late viral-clearance patients (positive SARS-CoV-2 RT-PCR >28 days). The bold line indicates the median, boxes indicate 25th and 75th percentiles and whiskers represent 1.5x IQR. Y-axis outcomes were log-transformed after adding 0.001. The dotted line represents the lower assay cut-off. (**A**) A comparison of IFN-γ concentration as measured by ELISA in supernatant of spike- and nucleocapsid-stimulated PBMCs between early and late viral-clearance patients at day 0 and 28. (**B**) SARS-CoV-2-specific reactive CD4+ T cells measured by AIM assay in early and late viral-clearance patients at day 0 and day 28. (**C**) SARS-CoV-2-specific reactive CD8+ T cells measured by AIM assay in early and late viral-clearance patients at day 0 and day 28. Zero inflated mixed models were used in statistical analysis. * P<0.05, ** P<0.01., *** P<0.001. Abbreviations: IQR: Interquartile range; AIM: activation induced marker

Supplementary Figure 6. Plot of SARS-CoV-2-specific B and T cell responses from patients with combined data available

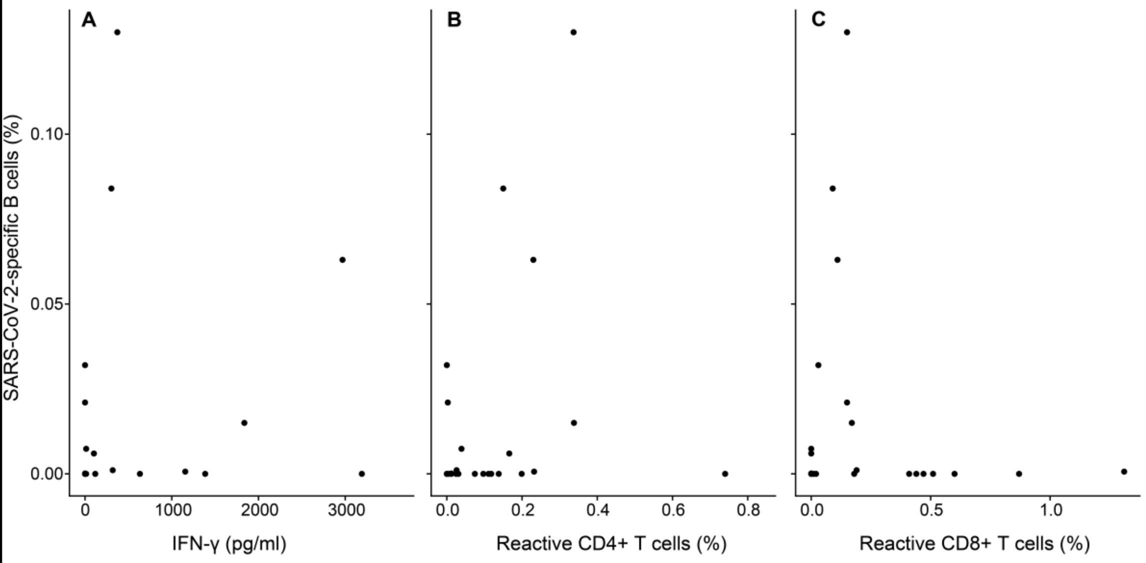

Supplementary Figure 6. SARS-CoV-2-specific B cell and T cell responses for all patients with combined data available. Ten day 0 and sixteen day 28 samples are plotted in each panel. (**A**) The IFN-γ concentration as measured by ELISA in supernatant of spike- and nucleocapsid-stimulated (X-axis) PBMCs against the proportion of SARS-CoV-2-specific B cells (Y-axis). (**B**) The proportion of SARS-CoV-2-specific reactive CD4+ T cells as measure by AIM assay (X-axis) against the proportion of SARS-CoV-2-specific B cells (Y-axis). (**C**) The proportion of SARS-CoV-2-specific reactive CD8+ T cells as measure by AIM assay (X-axis) against the proportion of SARS-CoV-2-specific B cells (Y-axis). AIM: activation induced marker.
